# High-Content Screening and Analysis of Stem Cell-Derived Neural Interfaces Using a Combinatorial Nanotechnology and Machine Learning Approach

**DOI:** 10.34133/2022/9784273

**Published:** 2022-09-14

**Authors:** Letao Yang, Brian M. Conley, Jinho Yoon, Christopher Rathnam, Thanapat Pongkulapa, Brandon Conklin, Yannan Hou, Ki-Bum Lee

**Affiliations:** Department of Chemistry and Chemical Biology, Rutgers University, The State University of New Jersey, Piscataway, NJ 08854, USA

## Abstract

A systematic investigation of stem cell-derived neural interfaces can facilitate the discovery of the molecular mechanisms behind cell behavior in neurological disorders and accelerate the development of stem cell-based therapies. Nevertheless, high-throughput investigation of the cell-type-specific biophysical cues associated with stem cell-derived neural interfaces continues to be a significant obstacle to overcome. To this end, we developed a combinatorial nanoarray-based method for high-throughput investigation of neural interface micro-/nanostructures (physical cues comprising geometrical, topographical, and mechanical aspects) and the effects of these complex physical cues on stem cell fate decisions. Furthermore, by applying a machine learning (ML)-based analytical approach to a large number of stem cell-derived neural interfaces, we comprehensively mapped stem cell adhesion, differentiation, and proliferation, which allowed for the cell-type-specific design of biomaterials for neural interfacing, including both adult and human-induced pluripotent stem cells (hiPSCs) with varying genetic backgrounds. In short, we successfully demonstrated how an innovative combinatorial nanoarray and ML-based platform technology can aid with the rational design of stem cell-derived neural interfaces, potentially facilitating precision, and personalized tissue engineering applications.

## 1. Introduction

Precise control of stem cell fates, such as adhesion, proliferation, differentiation, and apoptosis, is essential for diverse clinical applications such as stem cell therapy, disease modeling, and drug screening [1–4] . In humans, neural stem cells (NSCs) can be derived from various sources with distinct genetic backgrounds. These include NSCs that occur naturally in the hippocampus, and NSCs derived from pluripotent stem cells (e.g., human-induced pluripotent stem cell-derived NSCs (hiPSC-NSCs)) with or without mutations in functional genes [5, 6]. On the other hand, it has been known that different types of NSCs make their fate decisions in response to both soluble and insoluble biophysical cues that are mediated through the extracellular matrix (ECM) [7, 8]. Biophysical cues, such as geometrical, topographical, compositional, and mechanical properties of the ECM, have been increasingly regarded as crucial factors when designing stem cell-derived neural interfaces, such as nanofiber matrix-based NSC grafts, hydrogel-based spinal cord stimulation devices, as well as nanobiosensor-based neural probes [9–14]. Nevertheless, it is still unclear how the diverse neural behaviors of NSCs of different origins and genetic backgrounds could be dedicatedly regulated by varying ECM-mediated biophysical cues [15]. Specifically, micro-/nanotopographies of ECM, such as those present in protein fiber networks (i.e., collagen I/IV, fibronectin, and laminin), are abundant in many tissue types and known to profoundly affect adult neurogenesis through NSC cytoskeletal remodeling and modulation of biophysical signaling [16–20]. Researchers have thus made great efforts to understand the multifunctional roles of nanotopography on NSC fate decisions, encompassing the vast array of biophysical cues seen in natural ECM [21–27]. Yet, investigating the systematic relationship between the intricate mirco-/nanostructures of the ECM and the dynamic processes of fate determinations for the many types of NSCs is still a challenge that needs to be overcome. Addressing this critical barrier could not only provide a more complete view of NSC-ECM interactions but also influence the precision design of biomaterials used in stem cell-derived neural interfaces. Hence, there is an increasing need to develop a high-content screening (HCS) and high-content analysis (HCA) approach to facilitate a more comprehensive understanding of mechanisms associated with stem cell-derived neural interfaces.

**Figure 1:**
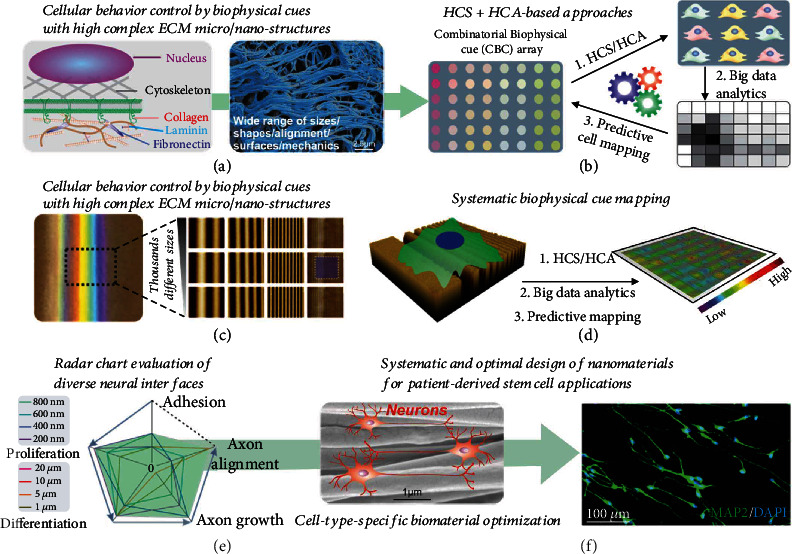
High-content screening of stem cell-derived neural interfaces using combinatorial nanoarray. (a) Schematic diagram (image on the left) and scanning electron microscopy (SEM of decellularized spinal cord) images showing the complexity and diversity of nanotopographies as biophysical cues for controlling cell behaviors. (b) Schematic diagram showing the HCS/HCA approaches to modulate the high complex biophysical cues and control stem cell fate. The approach would require: (1) high-content screening (HCS) and high-content analysis (HCA) of cells; (2) big data analytical approach for processing data generated from HCS and HCA approaches; (3) using the analyzed results to further predict the optimal biophysical cues and reoptimize the HCS and HCA method. (c) Generation of combinatorial nanoarrays with diverse micro-/nanostructures using dynamic laser interference lithography (DLIL), which could be used to screen biophysical cues by cell mapping (inset images are AFM images showing different sizes of micro-/nanoline structures). (d) Systematic biophysical cue mapping combined with machine learning-based big data analysis for the generation of predictive maps of nanotopography regulated stem cell fates. (e and f) Combinatorial nanoarray-based cell screening generate cell fate radar charts (e) for the assessment of biophysical cues for regulating neural stem cell behaviors and for predicting optimal nanofiber biomaterial structures (SEM image on the left is showing an optimal nanofiber structure predicted for axonal alignment) for guiding axonal alignment and other types of cell behaviors (immunostaining image on the right is showing the neuronal staining of neurons differentiated from adult stem cells) (f).

To address the above issues, herein, we developed a cell-type-specific combinatorial nanoarray and machine learning (ML)-based HCS and HCA method to analyze and predict biomaterial-regulated stem cell fate decisions [Figures 1(a) and 1(b)]. Remarkably, we could generate large-scale combinatorial nanoarrays encompassing thousands of patterned micro-/nanostructures on a single substrate with a wide size range (from 100 nm to 20 *μ*m) using our recently developed dynamic laser interference lithography (DLIL) [Figure 1(c)]. Although combinatorial nanoarrays have been constructed using conventional nanolithography techniques (e.g., electron beam lithography), they are typically on a smaller scale with fewer distinct structures [22, 24, 28, 29]. On the contrary, the combinatorial nanoarrays we constructed could serve as a diverse platform for investigating stem cell-derived neural interfaces. The challenges in tracking, correlating, and projecting many topographies with various types of stem cell behaviors are another obstacle that impedes the high-throughput investigation of neural interfaces [30]. To address this challenge, we combined a ML-based HCA method with the combinatorial nanoarray to map substrate topography-directed neural stem cell fate decisions more precisely, such as adhesion, proliferation, differentiation, axonal growth, and axonal alignment, across a wide range of topographies [Figure 1(d)]. Furthermore, while individual arrays with distinct nanotopographies have been used to evaluate neural differentiation, there are still significant challenges in realizing the predictive design of optimal biomaterial structures in a cell-type-specific manner [Figure 1(e)]. As such, we further evaluated our combinatorial nanoarray-based platform in the cell-type-specific neural interface design that could be useful for precision tissue engineering. Specifically, by screening optimal nanofiber structures to promote axonal alignment [Figure 1(f)], we identified stark differences among NSCs derived from the human hippocampus (adult NSCs), hiPSC-NSCs, and autism patient- (with MeCP2 mutation) derived hiPSC-NSCs, in terms of their responses to micro-/nanotopographies. Results from these comprehensive screening studies were further validated using conventional nanofiber-based stem cell differentiation assays and supported by genetic studies on the different neural stem cell types. In this manner, we could establish our combinatorial nanoarray as a flexible platform for investigating stem cell-derived neural interfaces and providing guidance for the cell-type-specific design of biomaterials for various personalized neural tissue engineering applications.

## 2. Results

### 2.1. Generation of Combinatorial Nanoarrays Using Dynamic Laser Interference Lithography

**Figure 2:**
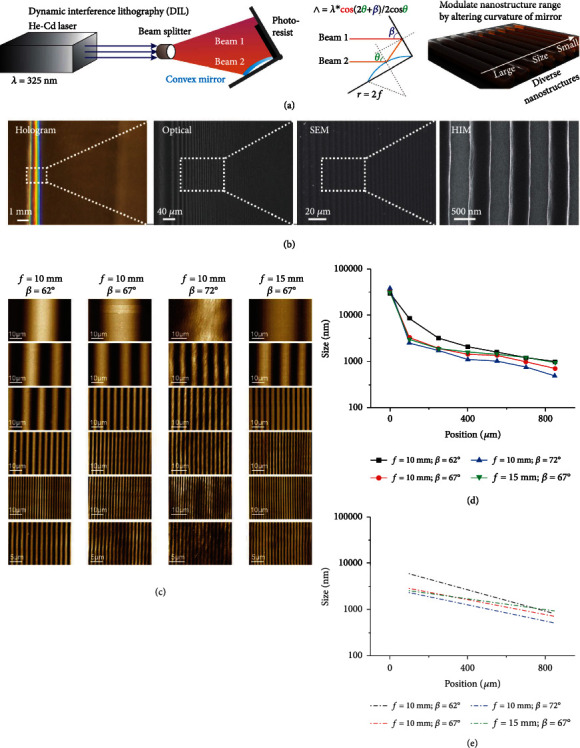
Tunable combinatorial nanoarrays with diverse micro-/nanostructures generated by dynamic interference lithography (DLIL). (a) A 325 nm helium-cadmium (He-Cd) laser generates two series of light beams (beam (1) directly derived from the original laser; beam (2): after reflection from the concave mirror that has spatially transformed beam angles) focused on a concave (Al)-coated mirror that produces many simultaneous interference patterns replicated on a photoresist-coated substrate to generate a gradient of micro-/nanoline patterns. Detailed optical simulation that derives the equation shown in the middle panel can be found in the supplementary information. (b) Optical microscopy, scanning electron microscopy (SEM), and helium-ion microscopy (HIM) all demonstrate a precisely fabricated gradient of topographical structures with strong hologram characteristic of nanotopographical features. (c and d) Atomic force microscopy (AFM) images (c) and quantifications of the micro-/nanostructures (d) characterizing the tunable combinatorial nanoarrays with different ranges of topographies. (e) Simulated curve of graph shown in *d*, where the log of the dimension of line patterns (*y*-axis) is nearly linear to position (*x*-axis) that allows reliable tracking of micro-/nanostructures in DLIL.

We first generated combinatorial nanoarrays encompassing diverse micro-/nanostructures using DLIL [Figure 2]. Micro/nanolithography techniques, including e-beam lithography, dip-pen nanolithography, mask-based photolithography, and 3D printing techniques, have been applied for fabricating micro/nanostructures in screening ECM-mediated biophysical cues [22, 28, 31, 32]. However, these approaches have been challenging to generate multi-scale and diverse structures (both micron-sized and nano-sized) in cost- and time-effective manners [Figure S1]. Previously we could generate large-scale homogenous arrays of nanostructures using an established laser interference lithography (LIL) technique [31], which further inspired us to develop DLIL by transforming the static interference field in a typical LIL setup into a vast array of non-periodic interference events with continuously changing interference angles. The process is high-throughput, with thousands of different structures formed within minutes, requiring no additional steps. [Figure 2(a)] [33]. After two-beam laser exposure on a photoresist-coated substrate, optical, electron, and atomic force microscopies indicated the successful formation of large-scale (1 cm by 1 cm) nonperiodic gradient arrays at high precision [Figure 2(b), S2, Table S1]. More specifically, this array contains a large combination of line patterns on a single substrate with precisely defined height, width, duty cycle, and periodicity. In addition, these patterns span a wide variety of structures with sizes from 20 *μ*m down to 100 nm that are highly relevant to both natural and synthetic ECMs and varying neural interfaces [17, 34]. Moreover, the range of micro-/nanostructures can be facilely tuned through modulation of the curvature of the interferometer [Figures 2(c)–2(e)]. Increasing the angle (*β*) between the static laser beam and the substrate from 62°, 67°, to 72° resulted in a narrower variety of micro-/nanostructure dimensions, whereas increasing the focal length (*f*, or curvature) of the convex interferometer resulted in a wide range of sizes [Figure S3]. In contrast, using Lloyd's mirror as the interferometer resulted in periodic arrays with singular nanostructures, which is consistent with previous literature [Figure S4]. For the rest of the study, we used *β* = 67° and *f* = 10 mm for the screening studies, as it not only provides a wide range of micro-/nanostructures, of which condition does not induce migration of NSCs across different structures.

### 2.2. High-Content Screening of NSC-Derived Neural Interfaces Using Combinatorial Nanoarray

**Figure 3:**
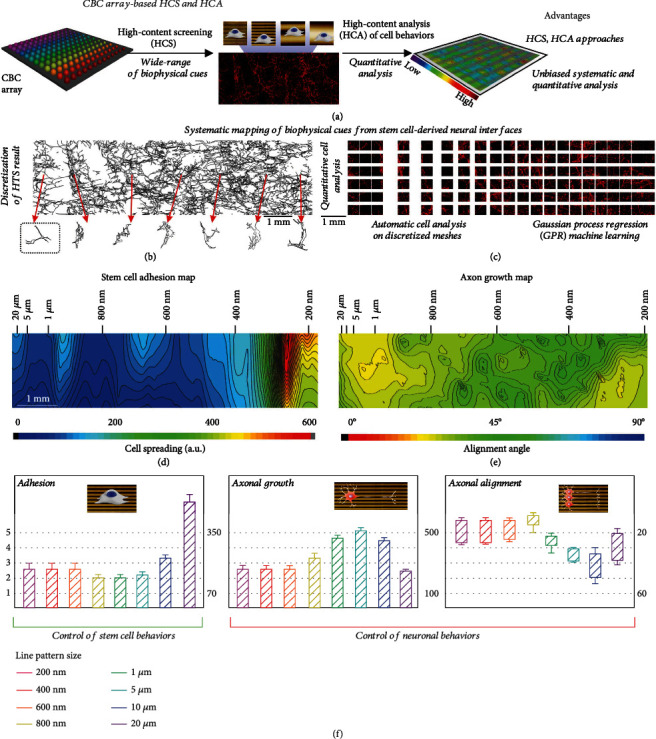
Systematic mapping of biophysical cues from stem cell-derived neural interfaces. (a) Schematic diagram showing the advantages of the CBC array-based high-throughput cell screening method for investigating biophysical cues. (b and c) Individual cell isolation- (b) and mesh-based (c) discretization methods for the analysis of immunostaining results (red: TuJ1 signal from neurons differentiated from adult NSCs) on the large-scale (around 1 cm in length) CBC array containing diverse micro-/nanostructures, which facilitates the generation of cell behavior maps through correlation between fluorescent signals with sizes of underneath micro-/nanostructures. (d and e) After discretization of the immunostaining images and correlating the cell behaviors (quantified by fluorescent signals) with micro/nanotopographies, cell behavioral maps, including adhesion (d, map on the left) and axon alignment (e, map on the right) could be generated. (f) By arbitrarily assigning five different levels for the scoring of each stem cell behavior, specific topographies for the five different cell behaviors could be evaluated based on analysis on cell maps. Immunostaining images and analytical approaches for each cell behavior of adult stem cells are detailed in supplementary information.

We next utilized the combinatorial nanoarray for the HCS and HCA of NSCs. Although the diverse (thousands of) micro-/nanostructures in the combinatorial nanoarrays could enable the systematic investigation and optimization of surface topographies of stem cell-derived neural interfaces, it becomes a challenge to track and analyze neural cell behaviors in response to the broad range of biophysical cues. This is further complicated by heterogeneous cellular responses, even when interfacing with the same set of biophysical cues [35]. For example, various neural cell behaviors, including adhesion, proliferation, differentiation of NSCs, as well as axonal alignment and growth of the differentiated neurons that are critical for many neurological studies, may favor different micro-/nanostructures, leading to difficulties for the rational design of stem cell-derived neural interfaces [Figure S5] [36–40]. For this purpose, we applied our combinatorial nanoarray and developed the analytical approaches for high-content screening (HCS) and mapping different neural cell behaviors [Figure 3(a)]. Specifically, as a proof-of-concept, adult NSCs were seeded on combinatorial nanoarrays at low densities, cultured in growth or differentiation media (see METHODS for details), then stained with biomarkers to investigate adhesion (actin staining via phalloidin), axonal alignment, growth, and differentiation (neuronal marker Tuj1). Combinatorial nanoarrays were all coated with laminin to promote their adhesion to the corresponding micro/nanotopographies and initiate the transmission of biophysical signaling of stem cells. Next, to establish quantitative relationships between neural cell behaviors and the underlying biophysical cues, post staining images were discretized into individual components to perform fluorescent intensity and cellular shape analysis (e.g., using Image J), and then correlated to the (*x*, *y*) positions of each discretized components [Figures 3(b) and 3(c), S6-S10, Table S2]. The distances *x* and *y* are defined as the horizontal distance from the initial point of exposure and the vertical distance from the bottom of the arrays, respectively, to the point of interest. We *hypothesized* that, by first correlating quantifiable neural cell behaviors to the position (*x*, *y*), followed by a correlation of these coordinates to the quantifiable biophysical cues as topographical features, we could establish the functional correlation between neural cell behaviors and biophysical cues on the stem cell-derived neural interfaces [Figures 3(d) and 3(e)]. However, an initial trial resulted in an entirely stochastic map between neural cell behaviors (e.g., adhesion area) and position (*x*, *y*) or nanotopography [Figure S11].

A plausible explanation for this observation is that the NSC-seeded combinatorial nanoarray contains cell-free regions that exhibit low behavioral values (e.g., no adhesion or neuronal differentiation) in response to corresponding biophysical stimuli, even though these cell-ECM interactions are absent. Although raising cell density to full confluence could partially address this difficulty, the complexities of cell-cell contacts will increase, and examination of neuronal morphological markers (e.g., axonal growth and alignment) will be more challenging. To address these challenges, we isolated discretized cell clusters using automatic cell recognition functions previously established (in CellProfiler and Image J, see METHODS section) to generate scattered maps of neural cell behaviors, followed by predictions of values between every two dots/units based on a Gaussian process regression (GPR) machine learning (ML) module in MATLAB [41–45]. To facilitate the data visualization, values on each neural cell behavior map were further arbitrarily assigned to 5 levels [Figure 3(f), S7-S10]. Briefly, NSC adhesion heavily favored the largest line pattern at 20 *μ*m, while line patterns from 200 nm to 10 *μ*m were comparable. NSC axonal growth peaked at 5 *μ*m line patterns and tapered off with both increasing and decreasing line widths. NSC axonal alignment favored nanometer-scale line patterns with suboptimal alignment in micron-sized line patterns. These mapping results represent some of the most comprehensive screenings of biophysical cues for regulating stem cell behaviors to the best of our knowledge. By integrating high-content analysis (HCA) and ML approaches into the combinatorial nanoarray, we could establish a new method for trackable, quantitative, and predictive biophysical cue mapping that is critically important for various stem cell-derived neural interfaces. As a result, our method dramatically reduces the amount of time and resources required to screen cellular behavior in response to specific biophysical inputs. Furthermore, it has the potential to be applied to any adherent cell type that is sensitive to the alteration of ECM topographies.

### 2.3. Cell-Type-Specific Evaluation of Neural Interfaces Using Combinatorial Nanoarrays

**Figure 4:**
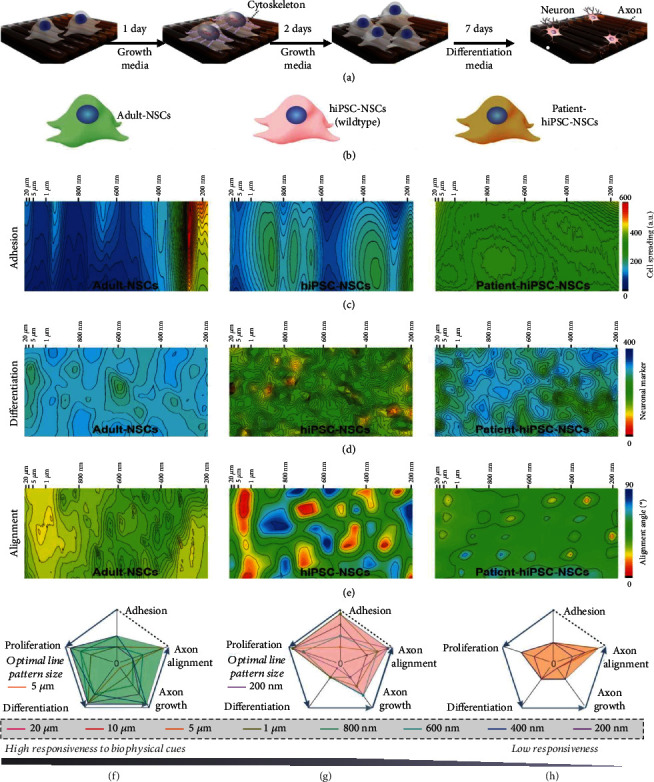
Combinatorial biophysical cue arrays identify cell-type-specific behaviors and elucidate mechanotransduction deficits while guiding optimized neural interface design. (a) Timelines for the generation of different cell maps from the three different stem cell types. (b) Schematic diagram showing the three different stem cell type screened by the combinatorial nanoarray-based cell mapping approach. (c–e) Size-dependent adhesion (c), differentiation maps (d) of adult-NSCs, hiPSC-NSCs, and patient-hiPSC-NSCs, and axonal alignment maps (e) of their differentiated neurons. Immunostaining images and analytical approaches used for generating these cell maps are detailed in supplementary information. (f–h) Radar charts generated from combinatorial nanoarray analysis comparing adult-NSCs (f), hiPSC-NSCs (g), and patient-hiPSC-NSCs (h), exhibiting a decrease in response to cellular behavior and biophysical cues within MeCP2-mutated patient-hiPSC-NSCs.

Considering the urgent need for the rational design of stem cell-derived neural interfaces in personalized tissue engineering, we first showcased our combinatorial nanoarray's ability to aid in the quantitative design of human patient-specific nanofibrous substrates [17, 18, 46–50]. For this purpose, three representative neural stem cell (NSC) types [human-induced pluripotent stem cell-derived neural stem cells (normal and healthy hiPSC-NSCs), autism patient-derived hiPSC-NSCs (patient-hiPSC-NSCs), and adult human NSCs (adult hNSCs) harvested from the human hippocampus region] were generated and utilized to investigate how diverse patient cells differentially respond to biophysical cues and regulate NSC fates and neuronal behaviors [Figure 4(a)] [51]. Human-induced pluripotent stem cells (hiPSCs) can be generated from patients having neurological disorders (e.g., autism and Parkinson's disease), thus maintaining the genotypic and phenotypic changes associated with these diseases [6, 52, 53]. Hence, hiPSC-NSCs could provide an excellent platform that closely mimics the human brain and central nervous system (CNS) for disease modeling and drug screening. Investigating the biophysical cues that control the differentiation of human-induced pluripotent stem cells (hiPSCs) into specific neural cell lineages and mature neural networks may also shed light on developmental biology and the step-by-step progression of neurodegenerative diseases [54, 55].

We first investigated the cell-type-specific modulation of hiPSC-NSC behaviors using our combinatorial nanoarray. Specifically, we mapped five key cell behaviors (adhesion, proliferation, differentiation of NSCs, axonal growth, and axonal alignment of differentiated neurons) of hiPSCs, patient-hiPSC-NSCs, and adult NSCs [Figures 4(b)–4(e), S7-S10, Table S3]. Strong evidence suggests that the lost cognitive functions in human patients with neurological disorders (e.g., autism) are associated with reduced sensitivity of neural cells toward their biophysical environment in the brain [56–59]. As such, we *hypothesized* that patient-hiPSC-NSCs would show impaired mechanosensitivity and reduced behavioral changes when exposed to the biophysical cues on the combinatorial nanoarray, as compared to hiPSC-NSCs and adult NSCs. Indeed, distinctively different patterns were observed in radar charts derived from the behavioral maps of the three NSC types. For example, patient-hiPSC-NSCs only showed slight differences in response to alterations of biophysical cues for almost all the crucial neural cell behaviors, including adhesion, proliferation, and differentiation [Figures 4(f)–4(h)]. On the other hand, the adhesion and proliferation scores shown on the radar charts of hiPSCs and adult NSCs exhibited significant differences in various biophysical signals. Additionally, our hypothesis on reduced mechanosensitivity in patient-derived hiPSC-NSCs was also supported by gene analysis of mechanotransduction-associated genes such as MeCP2, Grb2, Cdc42, Gap43, Ilk, Rac1, Src, Vcl, Ctnnb1, and RhoA as compared to the other two healthy NSC types [Figure S12, Table S3-S4]. In this way, the combinatorial nanoarray-based mapping method was applied to predict cell-type-specific modulation of cell behaviors and gene expression under various types of topographies in neural interfaces, thereby enabling the cell-type-specific design of neural interfaces for personalized tissue engineering applications.

### 2.4. Rational Design of Biomaterials through Combinatorial Nanoarray-Based HCS

**Figure 5:**
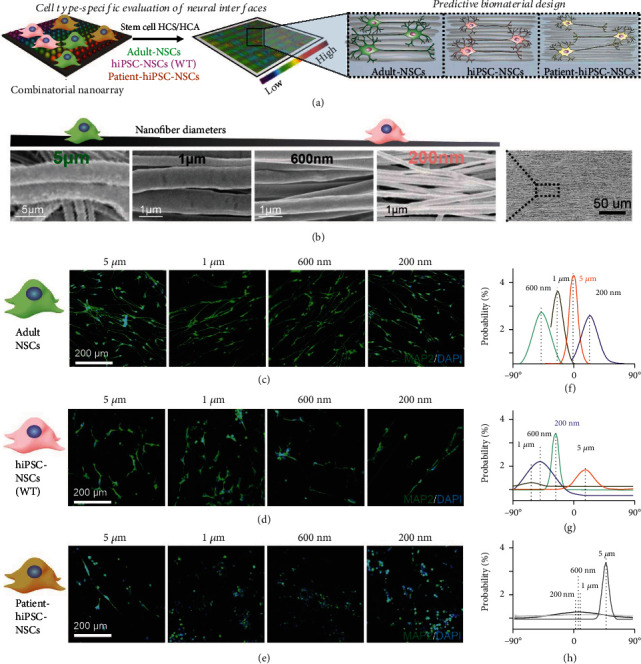
Rational design of cell-type-specific biomaterials as stem cell-derived neural interfaces using biophysical cue mapping. (a) Schematic diagram showing the cell behavior maps of 3 different types (adult-NSCs, hiPSC-NSCs, and patient-hiPSC-NSCs) for instruct the precise design of nanofibers in a cell-type-specific manner. (b) Scanning electron microscope (SEM) images of nanofibers with 4 different diameters. SEM image on the right is showing a zoomed-out view of a representative nanofiber. (c–e) Representative immunostaining images of axonal alignment of neurons differentiated from adult-NSCs (c), hiPSC-NSCs (d), and patient-hiPSC-NSCs (e). Green signal shows MAP2 as a neuronal marker for differentiated cells. (f–h) Graphs showing the orientation analysis (by Image J) of axonal alignment of neurons differentiated from adult-NSCs (f), hiPSC-NSCs (g), and patient-hiPSC-NSCs (h).

We next validated our design of topographies in neural interfaces using the conventional nanofiber biomaterial-based stem cell assays [Figure 5(a)]. Different cell lines express mechanotransduction genes at various levels and have different capacities to sense topographical cues [Figure S12]. As a result, topographical features of neural interfaces tailored to a specific cell could potentially enhance the viability of cell transplantation, cellular differentiation, and/or the overall therapeutic effect [18, 60]. To this end, we proved the rational design and engineering of neural interfaces in a cell-type-specific manner using the information generated from the combinatorial nanoarray-based cell mapping. As a proof-of-concept, we predicted and validated the optimal topographies for nanofiber biomaterial-mediated axonal alignment in a cell-type-specific manner. Specifically, based on our established radar charts of hiPSC-NSCs and adult NSCs, we identified the optimal biophysical cues (e.g., diameters of aligned 1D ECM nanostructures) that led to the most significant axonal alignment for adult-NSC as a proof-of-concept validation [Figure 4(e)].

We could then tailor nanofibers for various cell types based on the appropriate diameters we discovered through our mapping process. Using this design as a guide, we synthesized aligned nanofibers using a standard rotating drum electrospinning method, producing nanofibers with four distinct diameters (including the diameters that were projected to be ideal by our radar charts) [Figure 5(b), Table S5]. Next, stem cell differentiation assays using 3 different types of NSCs were performed on laminin-coated nanofibers for 7 days through growth factor withdrawal on these nanofiber matrices with varying topographies [Figures 5(c)–5(e), Table S4]. Immunostaining on a mature neuronal marker (MAP2) of differentiated NSCs allowed us to quantify the axonal alignment level of the differentiated neurons using Image J [Figures 5(f)–5(h)]. Image J's directionality module was utilized for the purpose of performing automatic quantification of axon alignment. Please note that, although neurons differentiated from the patient-derived hiPSC-NSCs also show a distinctive alignment peak, the alignment is overrepresented, as the directionality module did not recognize the dead/circular cell bodies well, which is commonly found in immunostaining images in [Figures 5(e)–5(1)]. Consistent with our results in the radar chart, in Figure 5 we show that axonal alignment in neurons differentiated from different cell types would favor different optimal nanofiber topographies. Based on Figures 5(c)–5(h), we found that neither all the nanofiber conditions nor all three cell types would always lead to nicely aligned axons. In general, adult NSCs, after differentiating into neurons, showed the most significant alignment. However, neurons differentiated from hiPSC-NSC (wild-type) also showed reasonably nicely aligned axons at the condition of nanofiber with a diameter around 200 nm [Figure 5(c)–5(e)], respectively, which fits the estimation in the radar charts shown in Figures 4(f)–4(h). As such, we successfully validated our high-throughput cell screening and biophysical cue mapping approach for engineering the topographies of neural stem cell-derived neural interfaces in human patient cell-type-specific manners.

## 3. Discussion

Because biophysical signaling is so vital in the construction of stem cell-derived neural interfaces, as well as the regulation and development of these interfaces, it would be so critical to develop a high-content screening and analysis of the biophysical functions on the neural interface, which can further affect the studying neurodegenerative diseases, disorders, and potential treatments. Although a few methods have been tried to screen cellular responses to biophysical cues in order to gain a better understanding of the cellular effect of nanoscale and microscale ECM topography, the majority of these studies have concentrated on either mechanistic pathways (such as the Hippo pathway) or the search for a biophysical cue that can regulate a specific cellular behavior (e.g., differentiation and axonal growth). Therefore, reliable nanoengineering methods to design a platform capable of large-scale biophysical cue screening in both high-content screening (HCS) and high-content analysis (HCA) manner are currently limited, delaying future discoveries on biophysical cues needed for regulating cellular behaviors. In order to overcome these obstacles, we successfully developed multiscale combinatorial biophysical cue (CBC) nanoarrays that encompassed a wide range of micro-/nanostructures using dynamic laser interference lithography (DLIL). These CBC nanoarrays were then combined with machine learning-based analysis in order to screen stem cell-derived neural interfaces in a comprehensive manner. Based on the HCS/HCA results, we could validate how the systematic optimization of neural interfaces can be incorporated with material topographical features specific to hiPSC-derived NSCs, and patient-derived iPSC-NSCs with mechanotransduction deficits, and hippocampus-derived adult NSCs, all of which could be used for personalized regenerative medicine. In summary, our combinatorial nanoarray-based screening platform holds excellent potential in a wide range of biomedical applications from disease modeling to stem cell therapies and may thus enhance the treatment of various types of neurological disorders. In the future, it remains a critical task to check if the combinatorial nanoarray could be applied as an implantable device and prosthesis for helping neural regeneration. It is also vital to investigate combining various pattern forms into the screening, such as dots and pillars, because there is no one topography that would result in optimum regulation of all cell behaviors. This will rapidly expand the applications of our cell screening platform, while requiring further advancement of a machine learning algorithm. In addition, the effects of height on the size-dependent cell behavior maps remain to be investigated, despite being a challenging task for the current CBC array-based platform. Also, despite its great promise, it remains elusive for the current combinatorial nanoarray platform to analyze 3-D neuron-topography interactions. Therefore, it would be essential to explore novel nanofabrication approaches for generating in vivo-like 3-D topography interfaces for stem cell transplantation and other screening applications.

## 4. Materials and Methods

### 4.1. Experimental Design

The goal of the current study is three-fold. The first goal is to identify suitable combinatorial nanoarrays. In order to accomplish this objective, we modified the optical paths in the DLIL that we had constructed to generate a variety of combinatorial nanoarrays. We select the most optimum nanoarray based on the wide range of micro-/nanostructures while minimizing unspecific cell migration to prevent the migration effect on the cell screening result. The second goal is to establish analytical methods for evaluating stem cell fate control by a vast array of nanotopographies on the combinatorial nanoarray. The difficulties associated with error readings on positions where stem cells were not present, which leads to the false null output of stem cell behaviors, present a significant obstacle in analyzing a large number of cell-nanotopography interactions. This is a significant barrier that must be overcome. This is why we incorporate the GPR machine learning method. To validate our strategy, we compared the cell mapping results with and without machine learning. The third goal is to provide a cell-type-specific design of biomaterials using combinatorial nanoarray-based stem cell screening methods. We chose a well-established adult neural stem cell line and two iPSC cell lines (wild-type and patient-derived), as they are highly relevant in neural stem cell therapies, and they are all human origin and cannot be studied in animal models. The limitation of the study is that when evaluating stem cell fate control by different micro-/nanostructures, the assignment of different levels of stem cell behavior scores is arbitrary, as there has been no previous analytical approach similar to the current one. The comparison is still valid even though it was conducted using the same standards for all different types of stem cells.

### 4.2. Dynamic Laser Interference Lithography (DLIL)

Glass slides (Fischer Scientific) were cut into individual pieces with areas of 1 × 1 cm^2^ and cleaned by sonication in Triton X-100 (1 wt%) aqueous solution, 190 proof ethanol, and ultrapure water. The cleaned glass slides were then dried and further cleaned by flowing nitrogen gas. To make the glass hydrophobic with better adhesion to photoresist, hexamethyldisilazane (HMDS) was deposited into the glass through vapor deposition for 12 hours in a vacuum oven. Glasses were then spin-coated with negative (AZ2020, Microchemicals, Germany, 1 : 0.8 dilution using AZ®EBR solvent) or positive Photoresist (AZ1505, Microchemicals, Germany, 1 : 1 dilution) using a spin-coater (Laurell Technologies, USA). For the negative photoresist, the glass substrates were preheated at 120°C for 1 minute, then placed to sample holder proximal to the curved (in the case of DLIL) or noncurved (in the case of regular LIL) interferometry, followed by exposure to ultraviolet (UV) laser (wavelength: 325 nm; He-Cd laser from KIMMON KOHA Laser Systems, Japan). The glass substrates were directly exposed to UV laser after spin-coating in terms of positive PR. After UV exposure, glass substrates were heated at 120°C for 1 minute, followed by incubation to develop a solution for 5-15 seconds. A hologram should appear after the developing step.

### 4.3. Nanoarray Characterization

Phase images of nanoarrays on glass substrates were collected from a Nikon microscope (T2500 series). Complex nanostructures of nanoarrays were carefully characterized by Atomic Force Microscope (AFM, Park Systems, NX10 series, tapping mode), Helium-ion Microscopy (HIM, Carl Zeiss, Orion Plus), and Zeiss Field-Emission Scanning Electron Microscope (FESEM, 20 kV).

### 4.4. Cell Culture (Table S1)

Human neural stem cell line [RenCell, or Adult-NSCs from Millipore (SCC008)] was cultured based on the manufacturer's protocol with slight modifications. Specifically, 25 cm^2^ tissue culture plates were coated with Matrigel (Corning) for two hours at 37°C, then one million RenCell was seeded and cultured in a humidified incubator with 5% CO2. We performed experiments on cells with passage numbers between seven and ten. Growth media for RenCell contains 0.5% N2, 0.5% B27, 20 ng/mL basic fibroblast growth factor (bFGF) and 20 ng/mL epidermal growth factor (EGF) which are dissolved in DMEM:F12 basal media. hiPSC-NSC-WT, and hiPSC-NSC-Q83 were derived from WT fibroblast and RTT fibroblast (WT126 clone 8; and WT33 clone 1), respectively, based on previous protocols. hiPSC-NSCs were expanded in a proliferation media containing DMEM/F12 with Glutamax (Invitrogen), B27-supplement (Invitrogen), N2 (Stem Cells), and 20 ng/mL FGF2 (Invitrogen). The differentiation of hiPSC-NSC-WT and hiPSC-NSC-Q83 was initiated by the withdrawal of FGF2 in the growth media. Tissue culture vessels were treated with Matrigel (Corning) 1 : 200 dilution with DMEM (Invitrogen) at 37°C for one hour. Media was changed on a bi-daily basis during growth and differentiation.

### 4.5. Cell Screening on Combinatorial Nanoarrays

To perform stem cell differentiation screening, we placed glass slides with combinatorial nanoarrays (1D line combinatorial nanoarray) into 24-well plates and then seeded stem cells into individual wells at a cell density of 40,000 cells per cm^2^. Combinatorial nanoarrays were coated with Matrigel for two hours before the cell seeding. Stem cells are seeded in growth media for four hours to stabilize and adhere to the substrate, and then the differentiation was initiated by FGF2 withdrawal into the growth media. After 7 days, cells on combinatorial nanoarrays were fixed, and immunostaining staining on neuronal markers (TuJ1 and MAP2) was performed to acquire the neuronal differentiation and axonal alignment maps. For the adhesion, proliferation, and axonal growth screening, lower cell densities at 10,000 cells per cm^2^ were seeded on combinatorial nanoarrays to minimize complicated effects from cell-cell interactions. Cells were fixed 12-hour and 7-day after seeding for the adhesion screening and axonal growth screening, respectively. Afterward, immunostaining on cytoskeletal markers (Actin) and neuronal markers (TuJ1) are conducted to attain the adhesion and axonal growth maps. In the stem cell proliferation screening, the stem cells were first labeled with CFSE dye (dilution 1 : 1000, Thermo Fisher Scientific) in accordance with the protocols provided by the vendor. After that, the stem cells were seeded onto the combinatorial nanoarrays, and then they were continuously cultured in growth media for five days. We then fixed and stained the cells with nuclear marker DAPI (Thermo Fisher Scientific). Based on the ratio of the fluorescent intensities between CFSE and DAPI, we can then quantify the relative proliferation rate for the stem cells.

### 4.6. Gene Expression Analysis by QRT-PCR (Table S2)

Cells on the substrate were detached and extracted with RNAs using Trizol® followed by PCR and SYBR green-based analysis followed vendor protocols (Applied Biosystems).

### 4.7. Antibodies and Immunostaining (Table S3)

We conducted immunocytochemistry to study topography-regulated cell behaviors after fixation on the combinatorial nanoarrays. All fluorescence images were acquired using a Nikon T2500 inverted fluorescence microscope. The nucleus was stained with DAPI (1 : 500 dilution, Life Technologies) for 30 minutes and then washed with PBS three times. Cells were permeabilized with 0.1% Triton X-100 in PBS for 10 minutes, and non-specific binding was blocked with 5% normal goat serum (NGS, Life Technologies) in DPBS for 1 hr. at room temperature. In the differentiation and axonal development experiment, cells were stained with neuronal markers TuJ1 and MAP2 using a mouse monoclonal antibody against TuJ1 (1 : 200 dilution, BioLegend) and MAP2 (1 : 200 dilution, BioLegend) (1 : 300 dilution, Cell Signaling). After incubating overnight at 4°C in a solution of these antibodies in PBS containing 10% NGS and washing three times with PBS, the samples were incubated for 1 hour at room temperature in a solution of anti-mouse secondary antibody labeled with Alexa Flour 568 (1 : 100, Life Technologies) in PBS containing 10% NGS, and washed with DPBS three times after that. In the adhesion assay, cells were stained with TRITC-labeled Phalloidin (1 : 100 dilution, Thermo Fisher Scientific) and DAPI (1 : 500 dilution, Life Technologies) for one hour, followed by DPBS washing. In the cell survival screening, live-dead kits (Thermo Fisher Scientific, L3224) were used following the protocol from the vendor. Briefly, we incubated cells with 1 *μ*m calcein (green color indicates live cell) and ethidium homodimer-1 (red color indicates dead cell) in the growth media for ten minutes and then imaged the large-scale substrates immediately.

### 4.8. Stem Cell Differentiation Assay on Aligned Nanofibers (Table S4)

To validate the high-throughput cell screening results, aligned polycaprolactone (PCL) nanofibers are synthesized by electrospinning technique. Varying diameters can be generated by controlling the parameters, including the concentration of PCL solution, speed of the rotating drum, and the distance between syringe and collector during electrospinning. Detailed parameters for synthesizing 200 nm (NF-200), 500 nm (NF-500), 1 *μ*m (NF-1000), and 5 *μ*m (NF-5000) sized aligned nanofibers can be found in Table S3. The nanofibers are transferred to a glass substrate using silicon glue. Afterward, the nanofibers were treated by oxygen plasma (Femto Science, Cute series) at 1.5∗10^−1^ torr for 60 seconds, sterilized by a UV lamp for one hour, and then coated with Matrigel for two hours. Stem cells were seeded to the nanofibers at a density of 40,000 cells per cm^2^ using identical procedures described in the LCT library-based stem cell screening. Four hours after seeding, FGF2 in the media was removed, and stem cells underwent neuronal differentiation for 7 days before being fixed and immunostained with neuronal markers (TuJ1 and MAP2).

### 4.9. Mapping Cell-Matrix Interactions Based on Cell Screening Results

For the stem cell adhesion screening assay, cytoskeletal structures of cells were stained with TRITC-labeled phalloidin across the large-scale substrate. Then the substrates were imaged by a Nikon T2500 inverted fluorescence microscope or ZEISS LSM 800 confocal microscope using automatic acquisition and stitching functions. Based on the stitched images, we built up cellprofiler pipelines that automatically identify fluorescent objects within the size ranges between 8 and 80 pixels (0.65 *μ*m per pixel). We found over 10,000 objects (cells) that were encoded with quantitative values of position, cell area, cell circularity, long-axis length, and short-axis length by utilizing this automatic method. Cell spreading is evaluated by the parameter of the cell area. By creating a function between cell spreading (*Z*) and position (*X*, *Y*) for each specific object using OriginLab or MATLAB, the quantitative 3D contour map visualizing the cell spreading and adhesion was generated. Similarly, based on the reverse of cell circularity, we created a 3D contour map visualizing the cell polarization as well. On these maps, by correlating the cell behaviors of individual cells to the topographies at each specific position using the functions in Figure S2, we could establish a functional relationship between cell behaviors and 3D material structures.

Cells differentiated for 7 days were stained with mature neuronal marker MAP2 and imaged using the fluorescent microscope in the stem cell differentiation screening assay. We first confirmed the homogenous cell densities based on the nuclear staining (DAPI), then the large-scale stitched images were removed with a fluorescent illumination background and pixelized into 48 to 576 arrays of smaller image files using the Nikon NIS Element software package. Automatic batch analysis on the fluorescent intensities of these pixelized images was performed in the batch process functions in ImageJ (https://imagej.net/Batch_Processing). By establishing the function between the fluorescent intensities (*Z*) and the position (*X*, *Y*) of each pixelized array, contour maps visualizing neuronal differentiation were generated.

To create an axonal growth map from the stem cell differentiation screening, we used the NeuronJ plugin in the ImageJ software package. In the large-scale stitched neuronal differentiation image, axons were stained by the red color from the immunostaining on the TuJ1 neuronal marker. We used the NeuronJ plugin to trace individual axons and then calculated each neuron's length based on the immunostaining image. Position (*X*, *Y*) of the starting point at each axonal tracing was recorded to correlate with substrate topographies. Axons that entangled each other or covered different ranges of topographies were not included in the analysis. By creating a function between axonal length (*Z*) and position (*X*, *Y*), we then built 3D contour maps visualizing topography-directed axonal growth.

For the cell proliferation map, cells were stained with the kit following the protocols from the ThermoFisher before their seeding onto the combinatorial nanoarray. After three days' proliferation in growth media (with bFGF for hiPSC-derived cell culture and bFGF/EGF for RenCell culture), cells were fixed and stained with DAPI. CFSE kit has been known to track the proliferation of cells based on their fluorescent intensities. Therefore, we used the Nikon NIS Ti Series microscope to image the substrate using a Stitch function followed by illumination background removal. After that, the CellProfiler pipeline that had been used to identify the cell morphologies described earlier was utilized to identify cells and automatically quantify the cell number and fluorescence intensity resulting from CFSE. By generating a function between the fluorescent intensities (*Z*) and position (*X*, *Y*) of each object (cell) identified by CellProfiler, contour maps visualizing the proliferation were then generated.

The Orientation J plugin in the Image J software package was used to analyze the stem cell differentiation results on the aligned nanofibers. In the study on neuronal differentiation from individual substrates, cells were stained with neuronal markers such as TuJ1, MAP2, NeuN, and nuclear marker (DAPI), followed by automatic detection of neuronal morphology using ImageJ or Nikon Ti Element software. The percentage of neurons, intensity of neuronal marker expression, or the axonal lengths were quantified. These studies on individual substrates include the neuronal differentiation from RenCell and hiPSC-derived NSCs on the line structures generated by LIL and the neuronal reprogramming from BJ fibroblast on the hierarchical structures synthesized by combined LIL and photo-mask lithography.

After obtaining the data points of position (*X*, *Y*) and the values for cellular behaviors (*Z*), a Gaussian process regression (GPR) machine learning module (https://www.mathworks.com/help/stats/gaussian-process-regression-models.html) in MATLAB® was used to connect the mapped data points and convert them into a heat map by plotting the results in OriginLab®. Specifically, the output graph will be presented as isolated points without machine learning, and the biophysical cue patterns without cell-seeded will be false-presented with a value zero (no signal from cells). Through the machine learning module in MATLAB®, this error could be corrected by learning from the trend of existent isolated data points and predicting the values of cell behaviors on biophysical cue patterns where the cell does not exist. The sizes of line-shaped nanotopographies were correlated to cellular behaviors by position parameter *X*, as shown by the function in Figure S4 and directly labeled on the graphs in Figures 3 and 4, and Figure S8-S11.

### 4.10. Selection of Gpr for Analysis of Cell Maps

“Gaussian Process Regression machine learning models are supervised by definition in that they are regression models; specifically, we are trying to understand relationships between dependent and independent variables. In this investigation, the labels correspond to coordinates on the *X*-*Y* plane (the independent variables) that are connected to locations on the substrate, and the result on the *Z*-axis (the dependent variable) represents the value that is being interpolated. In this way, we can label the coordinates on the *X*-*Y* plane and provide the biological outcome on the *Z*-axis so the model can properly make predictions and interpolate how cells would respond given new coordinates. Unsupervised models would consist of clustering, in which group unlabeled data with respect to their similarities and differences, and association, typically utilized by recommendation services, were not employed in generating this model. When selecting a ML model, one has to take into account the advantages and disadvantages an algorithm provides and, four our application, a GPR model was the most reasonable. GPR models are most efficient in low dimensional space, meaning our two key features would not overburden the algorithm. Moreover, GPR can perform probabilistic regression to measure the predictive uncertainty but does so at the price of poor scalability in the data. Because our data sizes were limited and we did not have thousands of inputs; GPR is one of the few models that can generate decent predictions on our scale. In addition to optimally functioning on smaller data sets with low dimensionality, GPR models are also highly versatile as various kernel functions can be applied to capture the features present in the data and improved the model's ability to interpolate predictions during the regression process. We did not try other types of machine learning methods as GPR was ideal for our low dimensional data sets with fewer entries while many other algorithms require larger data sets than was available.”

### 4.11. Machine Learning on the Mapped Results

A supervised GPR machine learning (GPR-ML) model, generated in MATLAB R2019b, was adopted to our system to continuously predict the spatial arrangement of cells when subjected to various regimes of our substrates. The dataset was preprocessed by combining the *X* and *Y* spatial coordinates into an observation matrix while placing the corresponding *Z*-coordinate into a label vector where the indices of the observation matrix and the label vector were consistent with one another. After separating the observations from the labels, the hyper-parameters were optimized using a Bayesian optimizer to ensure the learning process was updated and modified at each new evaluation of the model. It has been reported that Bayesian optimizers are proficient at finding a global optimum for an objective function in a minimal number of evaluations when tuning hyper-parameters by maximizing an acquisition function that will determine the next value where the model should be evaluated. In our model, we used an expected improvement (EI) criterion for the acquisition function to evaluate regions where the model believed the objection function was low and regions where the uncertainty was high. This was accomplished to address the exploitation versus exploration tradeoff many scientists encounter when selecting an optimizer. Hence, it was possible to search local areas within the bounds of the optimizer without overexploiting one area and being trapped at a local minimum. After establishing the optimizer, the observation data was split into 90% training and 10% testing data to allow for hold-out cross-validation to determine the model's predictive accuracy. After generating the GPR-ML model, the maximum and minimum values of the *X* and *Y* coordinates were identified and placed into a 2D grid composed of 250,000 values that spanned the area bounded by the maxima and minima to serve as inputs. Finally, this matrix was given to the GPR-ML model to predict the corresponding *Z*-coordinate.

### 4.12. Statistical Analysis

Bars in the graphs are mean ± standard deviation. The one-way analysis of variance (ANOVA) with Tukey posthoc analysis was used for multigroup analysis. The sample number was labeled in the figure caption of each figure. When the sample number is over 10, individual data points were plotted in the bar graphs. Graphs plotting and statistical analysis were performed using OriginLab® or Excel.

## Data Availability

Data associated with the current manuscript is available from the authors at reasonable request.

## Supplementary Materials

Supplementary Materials Figure S1: advantages of DLIL for generating combinatorial nanoarrays. Figure S2: characterization of combinatorial nanoarrays. Figure S3: optical pathway for gradient interference lithography. Figure S4: nanoarrays generated by conventional laser interference lithography .Figure S5: a schematic diagram showing the importance of ECM in neurobiology. Figure S6: zoom-in image of Figures 3(b) and 3(c). Figure S7: timeline and cell analysis on the adult-NSC differentiation map. Figure S8: CBC array-derived neuronal differentiation maps. Figure S9: CBC array-derived axonal growth maps. Figure S10: CBC array-derived adhesion and proliferation maps. Figure S11: a stochastic cell adhesion map generated without machine learning. Figure S12: gene analysis on mechanosensitivity of patient-hiPSC-NSCs. Table S1: quantification summary of Figure 2(e). Table S2: stem cell differentiation protocol and reagents. Table S3: primers used in qRT-PCR. Table S4: immunostaining protocols. Table S5: conditions for synthesizing aligned nanofibers.
