# Supplementary material for: High-Content Screening and Analysis of Stem Cell-Derived Neural Interfaces Using a Combinatorial Nanotechnology and Machine Learning Approach
Source: Research (Wash D C). 2022 Sep 14;2022:9784273. doi: 10.34133/2022/9784273 (PMC9513834; doi:10.34133/2022/9784273)
Supplement: Supplementary Materials — Figure S1: advantages of DLIL for generating combinatorial nanoarrays. Figure S2: characterization of combinatorial nanoarrays. Figure S3: optical pathway for gradient interference lithography. Figure S4: nanoarrays generated by conventional laser interference lithography .Figure S5: a schematic diagram showing the importance of ECM in neurobiology. Figure S6: zoom-in image of Figures 3(b) and 3(c). Figure S7: timeline and cell analysis on the adult-NSC differentiation map. Figure S8: CBC array-derived neuronal differentiation maps. Figure S9: CBC array-derived axonal growth maps. Figure S10: CBC array-derived adhesion and proliferation maps. Figure S11: a stochastic cell adhesion map generated without machine learning. Figure S12: gene analysis on mechanosensitivity of patient-hiPSC-NSCs. Table S1: quantification summary of Figure 2(e). Table S2: stem cell differentiation protocol and reagents. Table S3: primers used in qRT-PCR. Table S4: immunostaining protocols. Table S5: conditions for synthesizing aligned nanofibers. [file 9784273.f1.pdf]

SUPPORTING INFORMATION

High-Content Screening and Analysis of Stem Cell-Derived Neural Interfaces Using a Combinatorial Nanotechnology and Machine Learning Approach

*Letao Yang, Brian M. Conley, Jinho Yoon, Christopher Rathnam, Thanapat Pongkulapa, Brandon Conklin, Yannan Hou, and Ki-Bum Lee**

Department of Chemistry and Chemical Biology, Rutgers University, the State University of New Jersey, Piscataway, NJ 08854, U.S.A.

CORRESPONDENCE:

Prof. Ki-Bum Lee

E-mail: kblee@rutgers.edu

Website: https://kblee.rutgers.edu/

TABLE OF CONTENTS

SUPPLEMENTARY FIGURES (Page 3-13)

FIGURE S1 A diagram comparing conventional fabrication methods for combinatorial nanoarray

FIGURE S2 Characterization of combinatorial nanoarrays

FIGURE S3 Demonstration of the optical pathway for gradient interference lithography

FIGURE S4 Homogeneous singular nanoarrays generated by conventional laser interference lithography

FIGURE S5 A schematic diagram showing the importance of ECM in neurobiology

FIGURE S6 Zoom-in image of FIGURE 3b and 3c

FIGURE S7 Timeline and cell analysis on the adult-NSC differentiation map

FIGURE S8 CBC array-derived neuronal differentiation maps of adult-NSC, hiPSC-NSCS, and patient-hiPSC-NSCs

FIGURE S9 CBC array-derived axonal growth maps of adult-NSC, hiPSC-NSCS, and patient-hiPSC-NSCs

FIGURE S10 CBC array-derived adhesion and proliferation maps of adult-NSC, hiPSC-NSCS, and patient-hiPSC-NSCs

FIGURE S11 A stochastic cell adhesion map generated without GPR machine learning

FIGURE S12 Gene analysis reveals the impaired mechanosensitivity of patient-hiPSC-NSCs

SUPPLEMENTARY FIGURES (Page 14-15)

TABLE S1 Quantification summary of FIGURE 2e

TABLE S2 Stem cell differentiation protocol and reagents

TABLE S3 Primers used in qRT-PCR

TABLE S4 Immunostaining protocols

TABLE S5 Conditions for synthesizing aligned nanofibers

SUPPLEMENTARY FIGURES

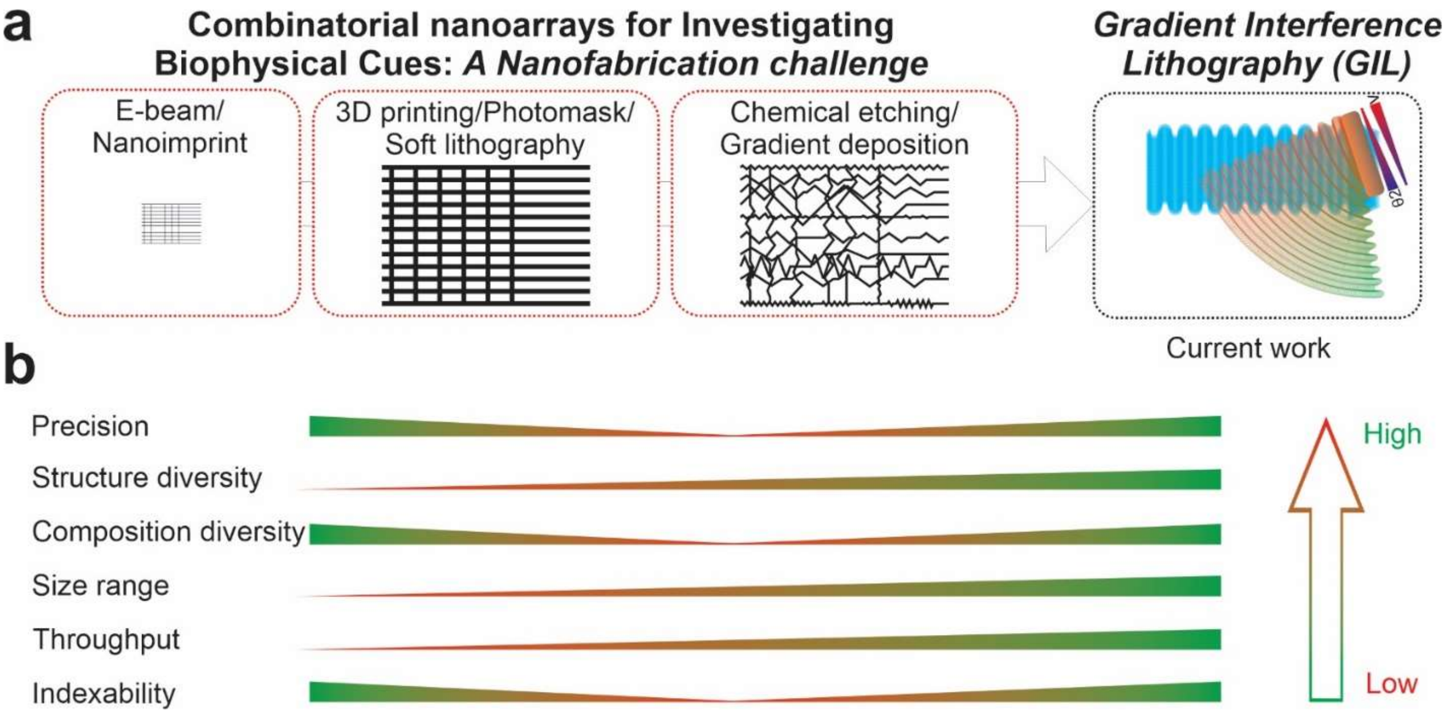

FIGURE S1. **A diagram comparing conventional nanofabrication methods for combinatorial ECM libraries.** Taken together, this scheme highlights the advantages of our developed dynamic interference lithography (DIL) in terms of high precision, high structural and compositional diversity (different shapes and alignment), wide size ranges, high throughput fabrication (mask-free) with programmable synthesis, and indexable nanostructures, which are all desired for the systematic investigation of ECM-cell interactions and biophysical cues. Please note that compared to LIL previously applied for cell-matrix studies, dynamic laser interference lithography (DLIL) generates considerably more complex patterns, from thousands to millions of different sizes and geometries, in a single lithography process. At the same time, LIL only produces one homogeneous nanostructure at one time. Therefore, DLIL demonstrates clear advantages over LIL in systematically investigating biophysical cues.

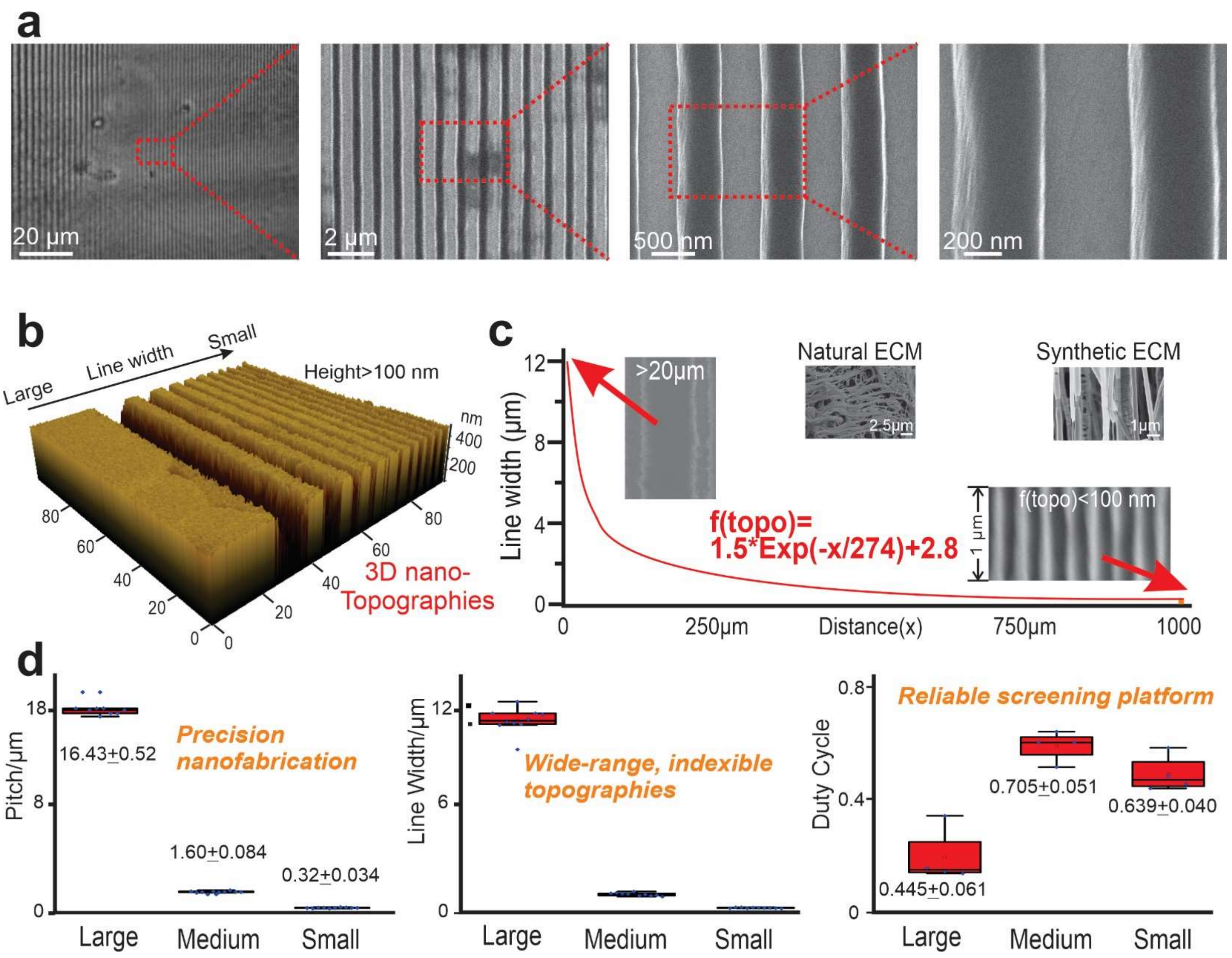

FIGURE S2. **Characterization of combinatorial nanoarrays.** **(a)** Optical (image on the left) and helium-ion microscopy (HIM, images on the right) characterizations of the combinatorial nanoarrays fabricated by DIL at high precision. **(b-c)** Atomic force microscopy (AFM) details a highly uniform height profile across the nano/micro line patterns with hierarchal line widths ranging from 100nm to 20 um. Inset images in d are SEM characterization of natural ECM. **(d)** Quantification of size characteristics from gradient nano/micro line patterns demonstrating that DLIL can generate precise nanofabrication, a wide range of line widths, and tunable duty cycles.

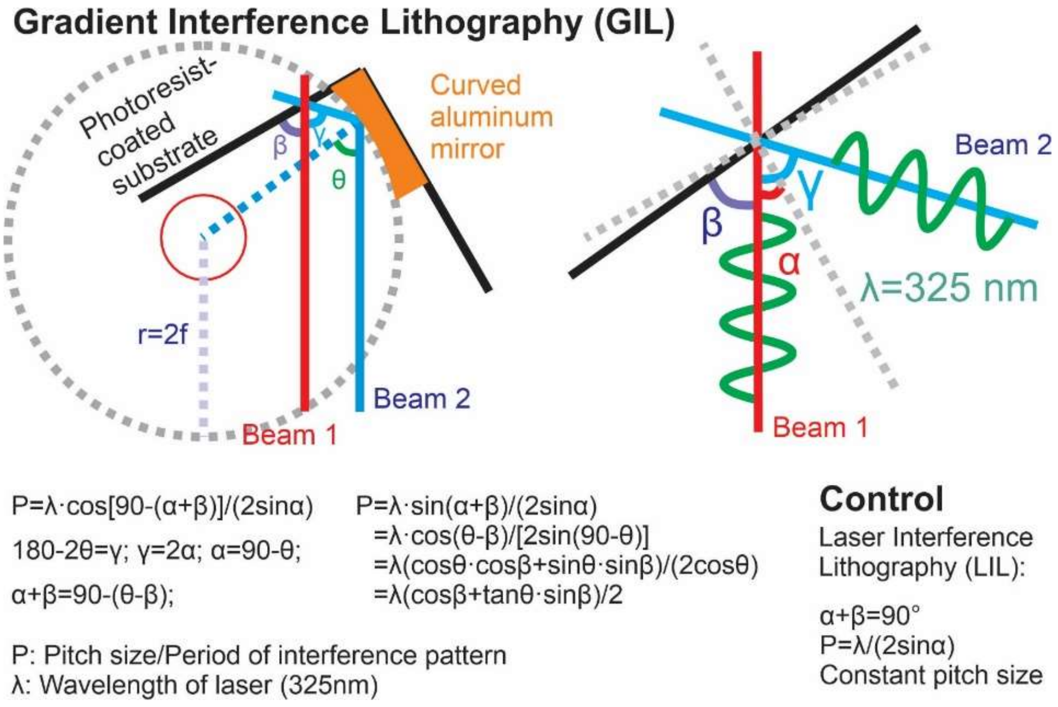

FIGURE S3. Optical analysis of DLIL set-up. In traditional LIL based on Lloyd's mirror interferometers, a mathematical representation of the interferometric pattern formation can be written as $\Lambda=\lambda/(\sin\eta_0+\sin\eta')$ where Λ = periodicity, λ=wavelength, and η_0,η' = angles normal to the exposed surface of Beam 1 (from the original laser) and Beam 2 (reflected from the mirror). Because Lloyd's mirror in the interferometer is plane-shaped, η' is identical across the mirror and photoresist surface, leading to the formation of singular nanostructures after laser exposure. In DLIL, however, the curved mirror will differentially reflect and transform the incident Beam 2 at varying angles, resulting in the formation of gradient nanoarrays with a wide range of trackable micro/nano-structures.

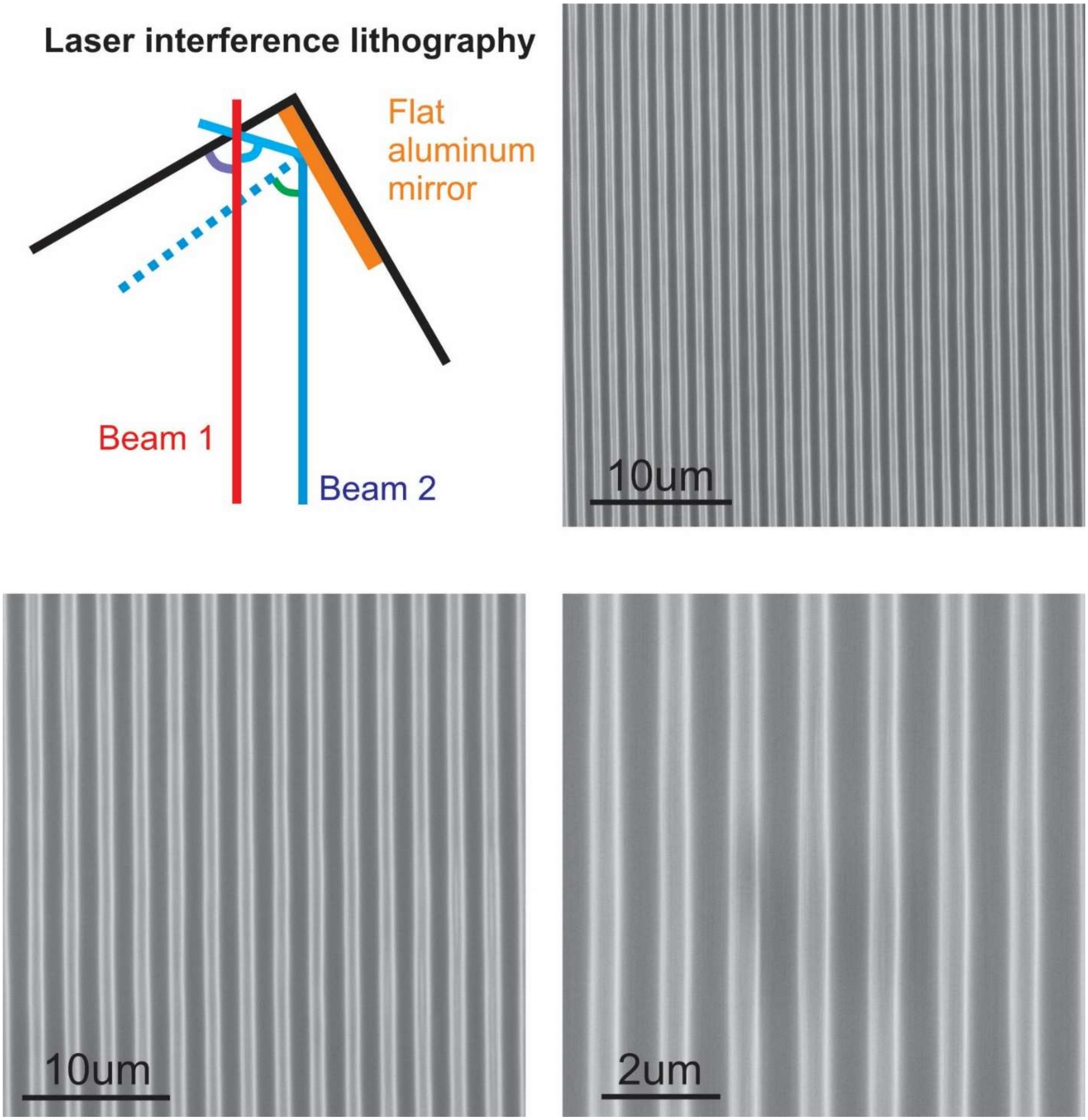

FIGURE S4. **Control substrate with homogeneous and singular nanostructures generated by conventional laser interference lithography (LIL).**

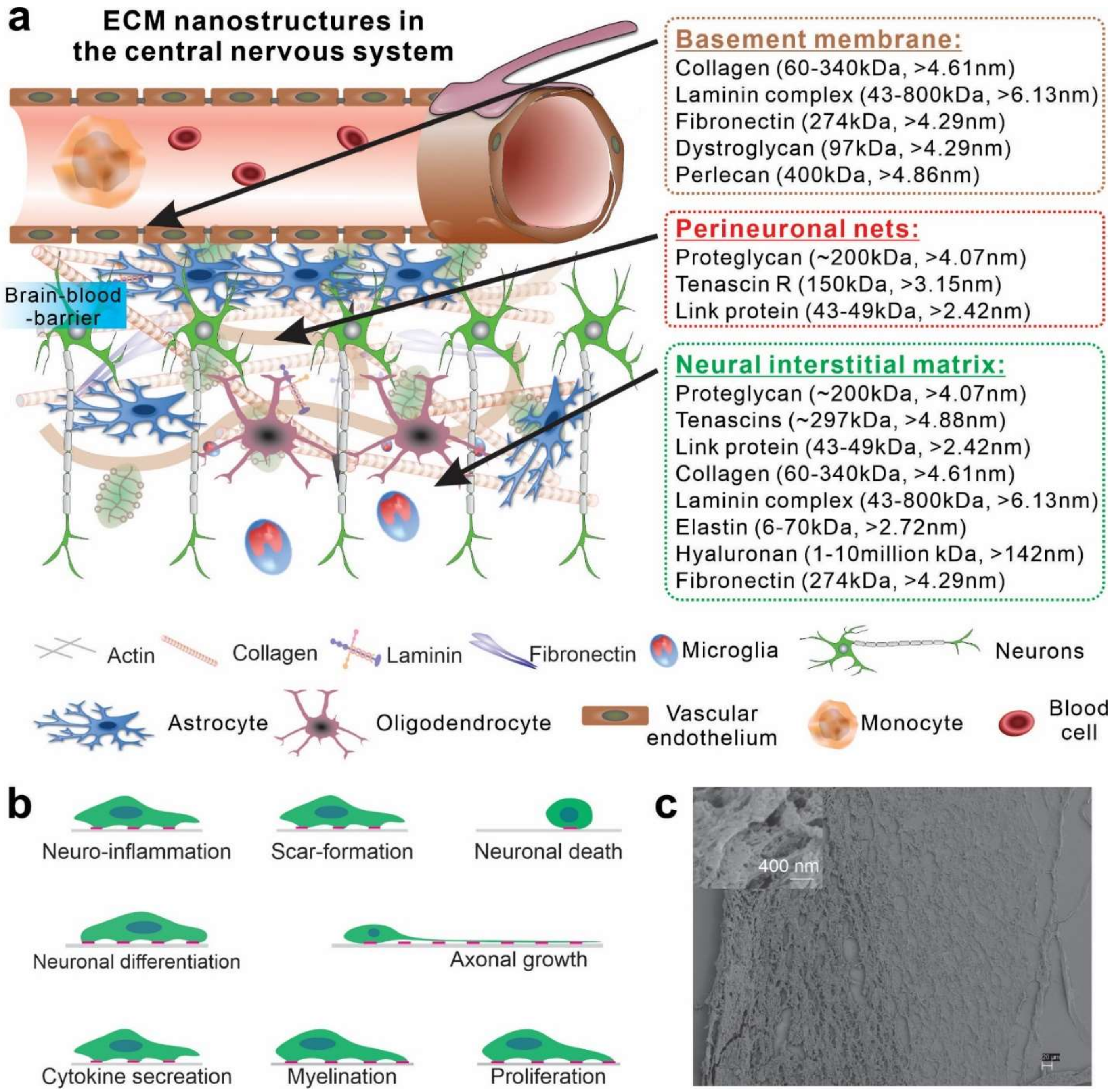

FIGURE S5. **A schematic diagram showing the importance of ECM topography (e.g., sizes and shapes) in neurobiology.**

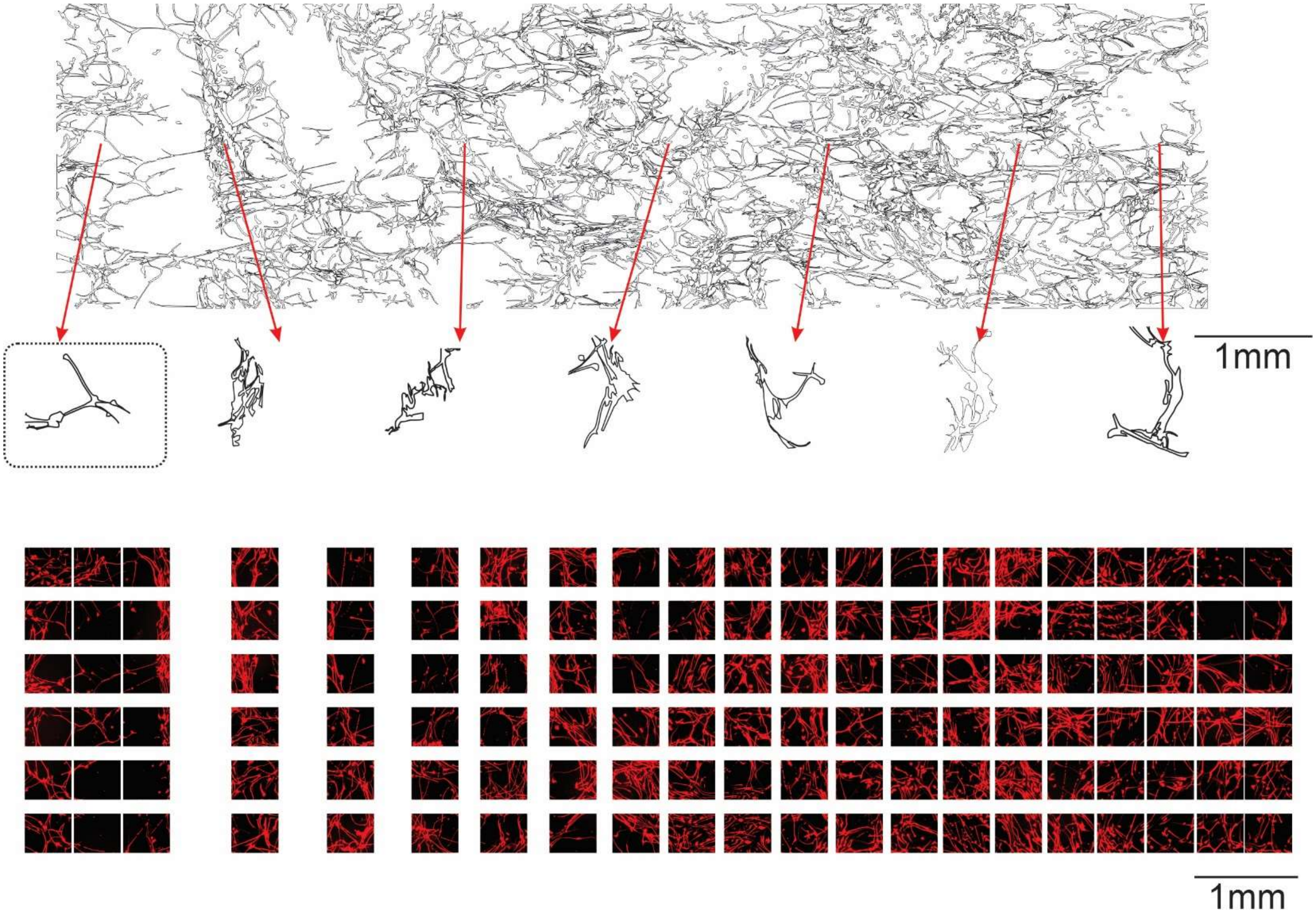

FIGURE S6. Zoom-in image for panel b and c in FIGURE 3.

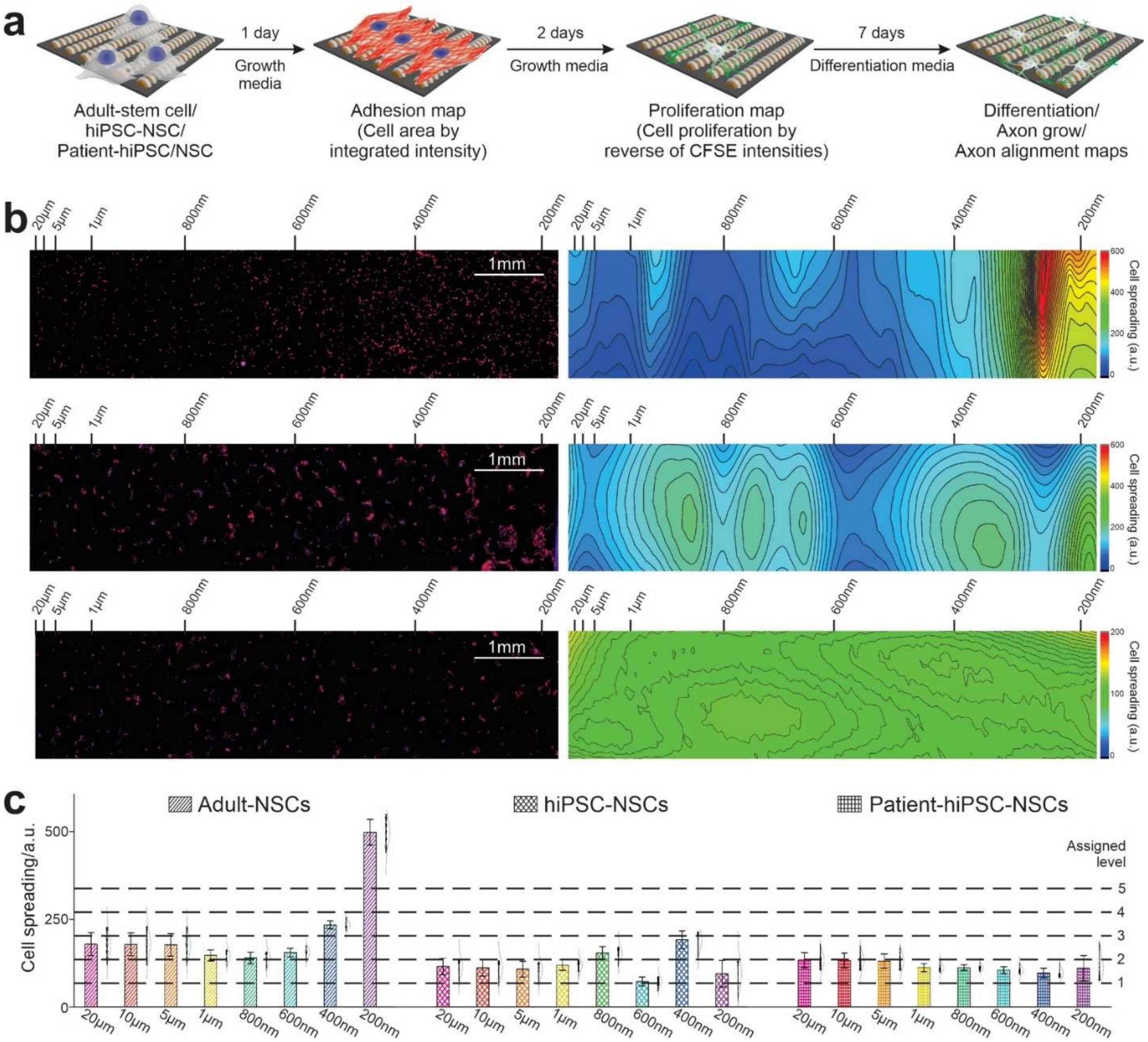

FIGURE S7. Combinatorial nanoarray-based neuronal adhesion assay (**a**) and the generated adhesion maps (**b**) of adult-NSC, hiPSC-NSC, and hiPSC-NSC-Q83, as well as the quantifications at representative biophysical cues (sizes of the 1D line nanostructures, **c**). Immunostaining images on the left of panel **b** are from phalloidin staining (colored in red) on the actin of different NSC types cultured on CBC array for 3 days. Cell spreading was automatically calculated using CellProfiler and plotted against the position of each cell cluster in OriginLab.

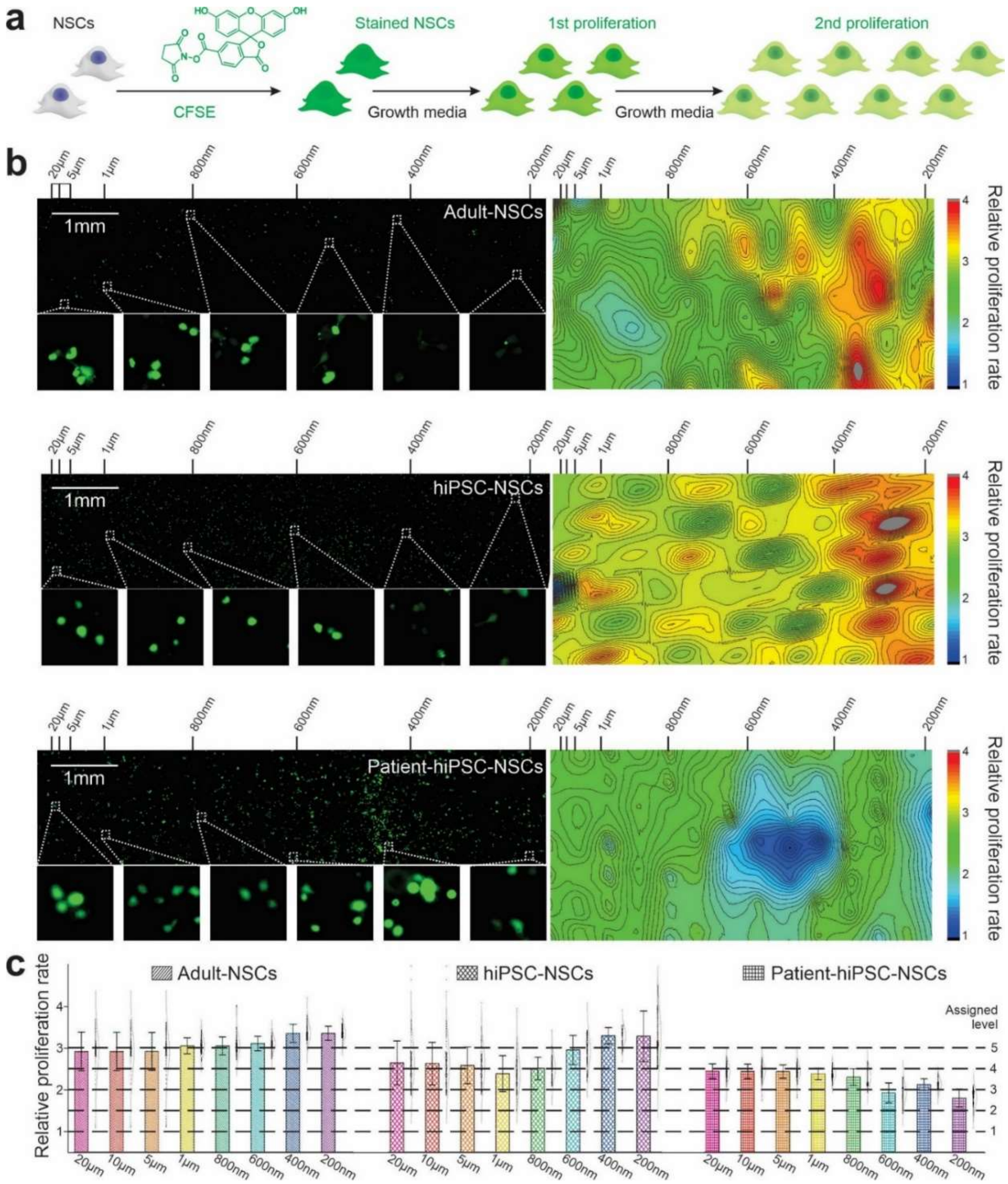

FIGURE S8. Combinatorial nanoarray-based neuronal proliferation assay (schematic diagram shown in **a**) and the generated proliferation maps (**b**) of adult-NSC, hiPSC-NSC, and hiPSC-NSC-Q83, as well as the quantifications at representative biophysical cues (sizes of the 1D line nanostructures, **c**). Immunostaining images on the left of panel **b** are from CFSE® staining (colored in green) on the actin of different NSC types cultured on the CBC array in proliferation media for 3 days. The quantification of proliferation rate is based upon the CFSE® staining protocol, and higher fluorescence indicates a lower proliferation rate, as each cell doubling event will result in the splitting of CFSE dye concentration into two separate cells.

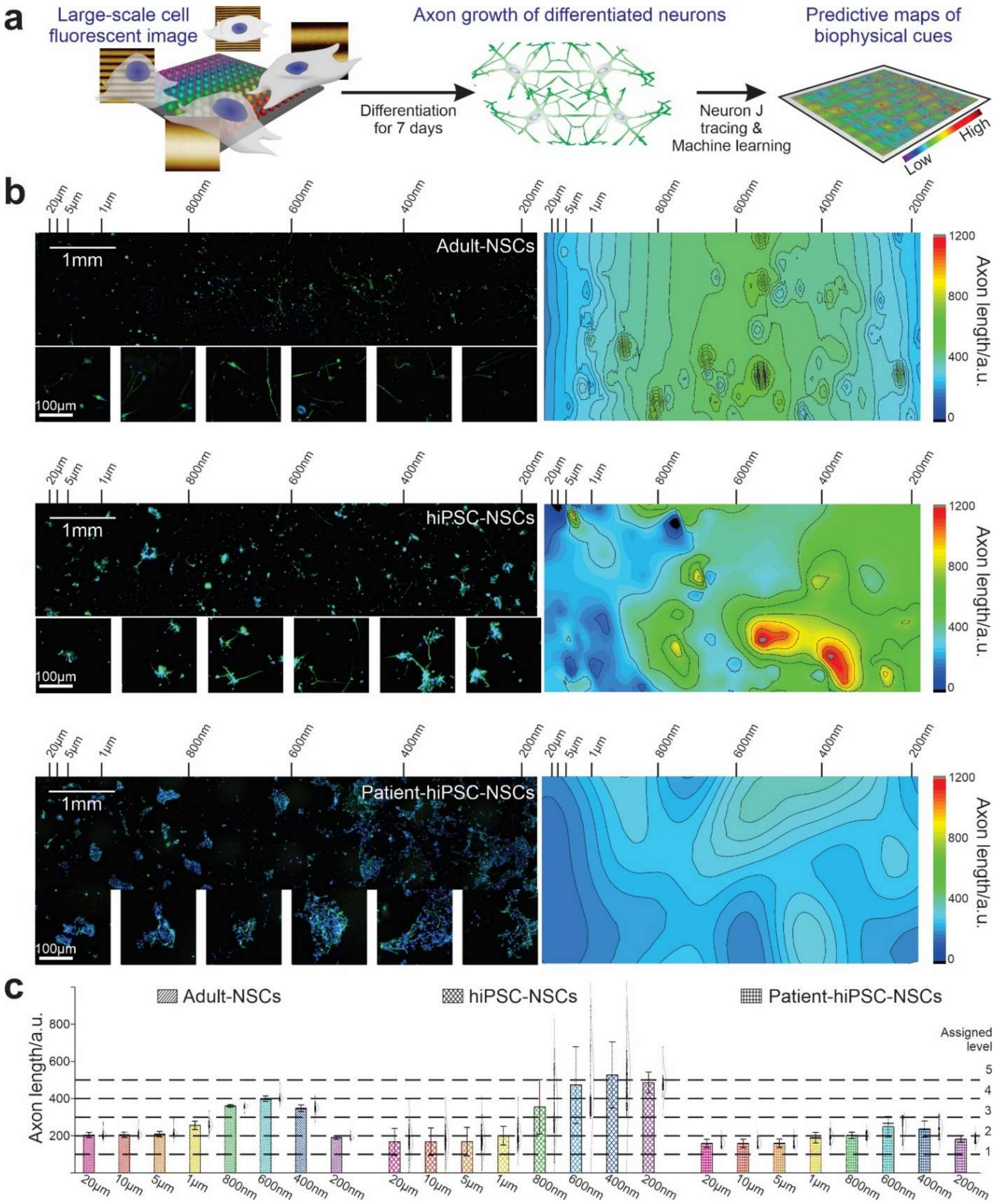

FIGURE S9. Combinatorial nanoarray-based axonal growth assay (schematic diagram shown in **a**) and the generated axonal growth maps (**b**) of adult-NSC, hiPSC-NSC, and hiPSC-NSC-Q83, as well as the quantifications at representative biophysical cues (sizes of the 1D line nanostructures, **c**). Immunostaining images on the left of panel **b** are from immunostaining of neuronal markers (MAP2, colored in red) on the actin of different NSC types differentiated on the CBC array for 7 days. The axon length map was obtained by the NeuronJ module in the Image J software.

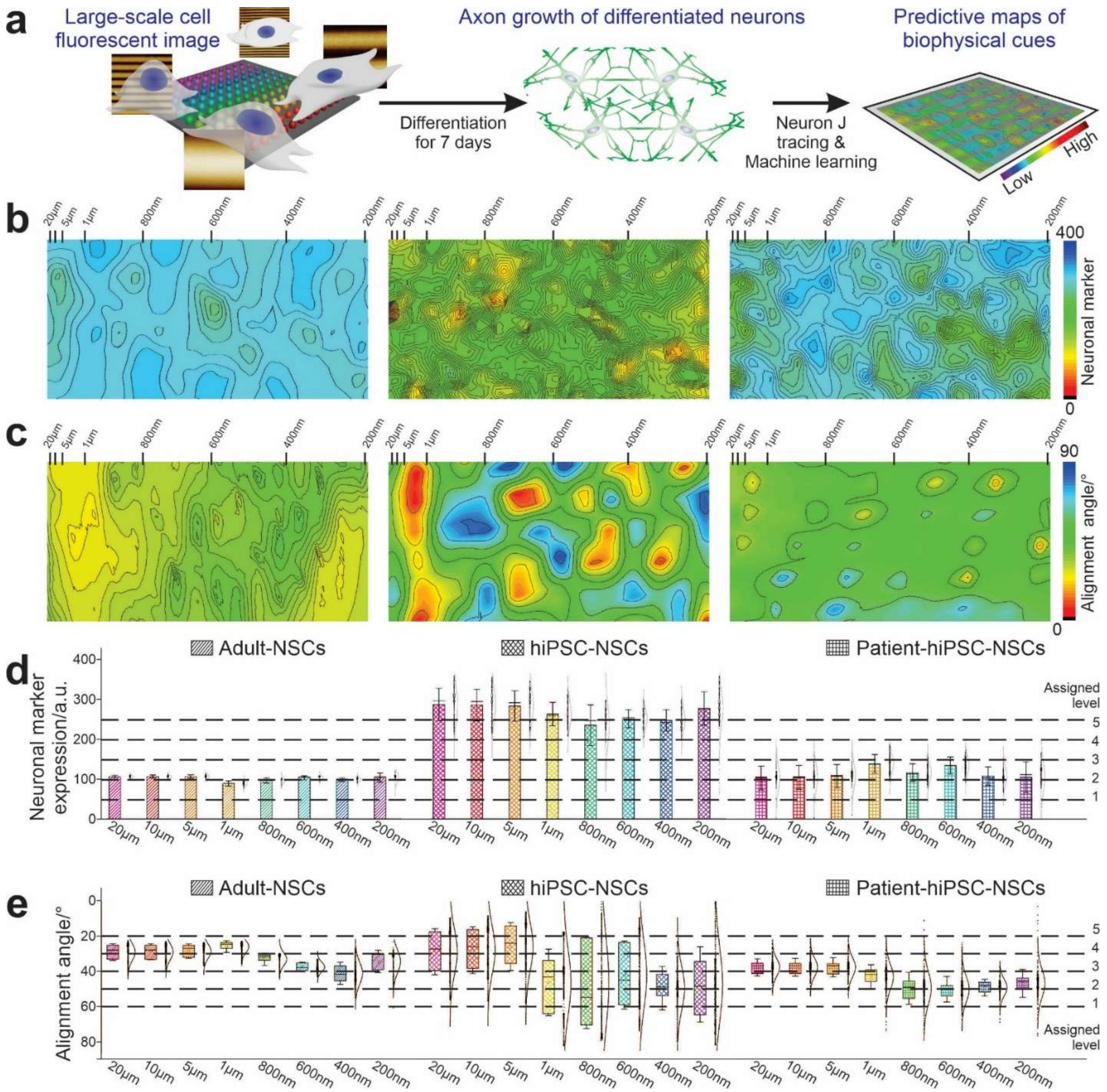

FIGURE S10. Combinatorial nanoarray-based neuronal differentiation and axonal alignment assay (schematic diagram shown in **a**) and the generated differentiation (**b**) and axonal alignment maps (**c**) of adult-NSC, hiPSC-NSC, and hiPSC-NSC-Q83, as well as the quantifications (**d-e**) at representative biophysical cues (sizes of the 1D line nanostructures). The expression of neuronal marker and cell alignment angle were quantified by the fluorescence intensity and plotted against the location of corresponding cell regions using Nikon software and Neuron J module in the Image J software, respectively.

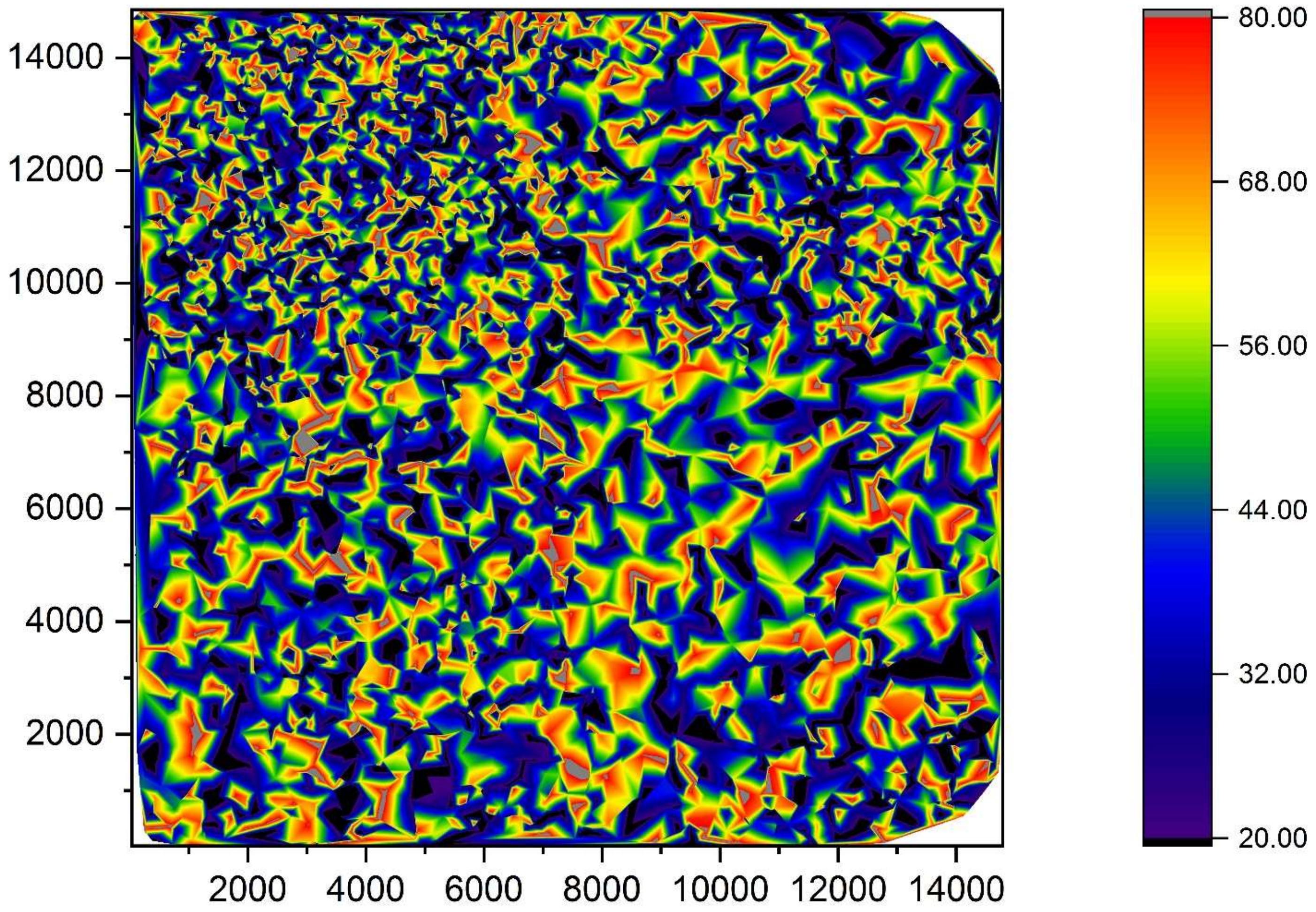

FIGURE 11: An exemplary adhesion orientation map generated without a GPR machine learning algorithm. The map displays a stochastic pattern resulting from the presence of cell-free regions in the NSC-seeded CBC array that randomly exhibits low behavior values (e.g., no adhesion or neuronal development) at the corresponding biophysical cues despite the absence of cell-ECM interactions.

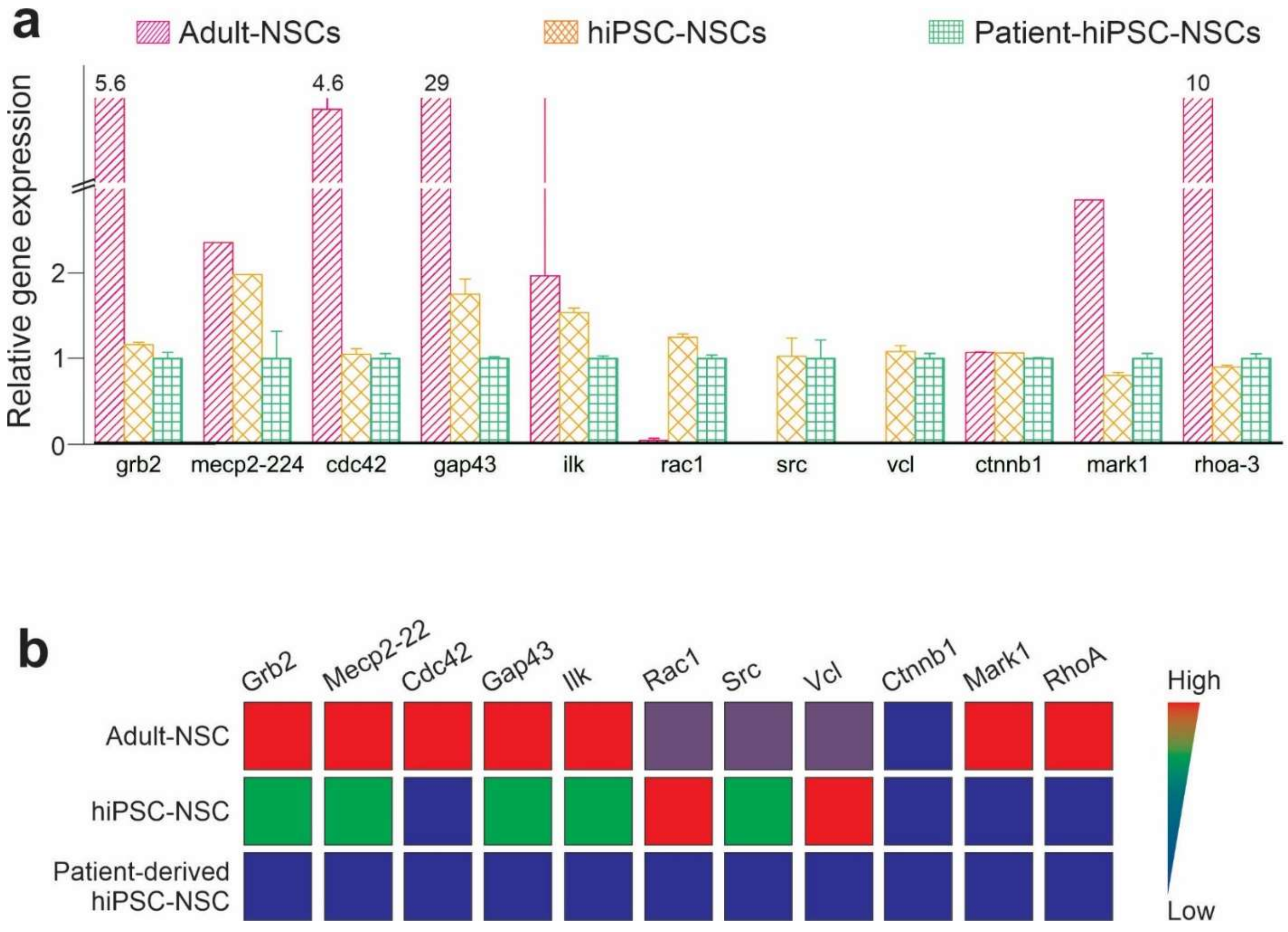

FIGURE S12. Bar graph (a) and heatmap (b) summarizing qRT-PCR analysis on the mechanotransduction-related gene expression from the 3 different NSC lines (adult NSCs, hiPSC-NSC-WT, and hiPSC-NSC-Q83). The result in the bar graph is normalized to the control gene GAPDH. Gene expression levels higher than 10 were cut-off for better visualization.

Condition	Equation	R-square
f=10mm; β=62°	log(y)=-0.001145*x+3.997298	0.93
f=10mm; β=62°	log(y)=-0.000814*x+3.621444	0.95
f=10mm; β=62°	log(y)=-0.000606*x+3.54164	0.95
f=10mm; β=62°	log(y)=-0.000882*x+3.532211	0.94

TABLE S1. Equation of simulated curves shown in FIGURE 2e. x indicates the distance from the starting line of the nanoarray (unit: μm); y indicates the size of line patterns (unit nm); f indicates the focal length of the curved mirror; β means the angle between the curved mirror and the original laser beam.

	RenCells	hiPSC-NSCs-WT	Patient-hiPSC-NSCs
**Substrate coating**	Laminin (Sigma Millipore CC095)	Laminin (Sigma Millipore CC095)/ Matrigel (Corning)	
**Proliferation media**	neural basal medium (Gibco) and DMEM/F12 (Gibco) (50:50 ratio) supplemented with 0.5 % N2 (Gibco), 0.5 % B27, and 20 ng/mL EGF and bFGF (Fibroblast growth factor-basic, PeproTech)	DMEM/F12 with Glutamax (Invitrogen), B27-supplement (Invitrogen), N2 (Stem Cells), and 20 ng per mL bFGF (Invitrogen)	
**Differentiation media**	Neural basal medium (Gibco) and DMEM/F12 (Gibco) (50:50 ratio) supplemented with 0.5 % N2 (Gibco), 0.5 % B27	DMEM/F12 with Glutamax (Invitrogen), B27-supplement (Invitrogen), N2 (Stem Cells)	

TABLE S2. Media formulations and reagents used in cell culture.

Gene	Forward	Reverse	Species
**MeCP2**	ACATCAGAAGGGTCAGGCTC	CCCTGCCCTGTAGAGATAGGA	Human
**Grb2**	CTGGGTGGTGAAGTTCAATTCT	GTTCTATGTCCCGCAGGAATATC	Human
**Cdc42**	CCATCGGAATATGTACCGACTG	CTCAGCGGTCGTAATCTGTCA	Human
**Gap43**	AACCTGAGGCTGACCAAGAA	GGGACTTCAGAGTGGAGCTG	Human
**Ilk**	TGGAACCCTGAACAAACACTC	AGCACCTTCACGACAATGTCA	Human
**Rac1**	ATGTCCGTGCAAAGTGGTATC	CTCGGATCGCTTCGTCAAACA	Human
**Src**	GACAGGCTACATCCCCAGC	CGTCTGGTGATCTTGCCAAAA	Human
**Vcl**	CCAAGATGATTGACGAGAGACAG	AGAGGTGAGTTGTAACACACGA	Human
**Ctnnb1**	CATCTACACAGTTTGATGCTGCT	GCAGTTTTGTCAGTTCAGGGA	Human
**RhoA**	AGCCTGTGGAAAGACATGCTT	TCAAACACTGTGGGCACATAC	Human
**Gapdh**	AGGAAATGAATGGGCAGCCGT	TAGCCTCGCTCCACCTGACT	Human

TABLE S3. Primers used in qRT-PCR experiments.

Markers	TuJ1	MAP2	CFSE
**Fixation protocol**	4% formalin, 10 minutes		N.A.
**Blocking buffer**	1% Triton X (Sigma Millipore), 0.5% Bovine serum albumin (Sigma Millipore), 0.5% goat serum (Sigma Millipore) in ultrapure water		N.A.
**Dilution buffer**	1% Triton X (Sigma Millipore), 1% Bovine serum albumin (Sigma Millipore), in ultrapure water		Culture media
**Primary antibody vendor**	Biolegend	Biolegend	Thermo Fisher Vybrant DyeCycle Green (V35004)
**Primary antibody dilution**	1:300	1:400	1:1000
**Secondary antibody vendor**	Biolegend	Biolegend	N.A.
**Secondary antibody**	594 nm Goat anti-mouse	488 nm Goat anti-rabbit	N.A.
**Microscope for imaging**	Nikon Ti series/ Leica LSM 800 confocal microscope		Nikon Ti series

TABLE S4. Protocols and materials used in immunostaining.

	200 nm	500 nm	1 µm	5 µm
**Voltage**	5kV	5kV	5kV	5kV
**Concentration (mass)**	1%	2%	5%	10%
**Rotating drum**	1000rpm	1000rpm	1000rpm	1000rpm
**Spinning distance**	10cm	10cm	10cm	10cm
**Spinning speed**	1.0mL/hour	1.0mL/hour	1.0mL/hour	1.0mL/hour

TABLE S5. Experiment parameters for electrospinning of aligned nanofibers.
